# Experimental Validation of Time-Explicit Ultrasound Propagation Models with Sound Diffusivity or Viscous Attenuation in Biological Tissues Using COMSOL Multiphysics

**DOI:** 10.3390/bioengineering12090946

**Published:** 2025-08-31

**Authors:** Nuno A. T. C. Fernandes, Shivam Sharma, Ana Arieira, Betina Hinckel, Filipe Silva, Ana Leal, Óscar Carvalho

**Affiliations:** 1 Center for Micro-Electro Mechanical Systems (CMEMS), University of Minho, 4800-058 Guimarães, Portugal; shivam0671@gmail.com (S.S.); ana.a.arieira@gmail.com (A.A.); fsamuel@dem.uminho.pt (F.S.); anaisabeleal@gmail.com (A.L.); oscar.carvalho@dem.uminho.pt (Ó.C.); 2 Department of Orthopaedic Surgery, William Beaumont Hospital, Royal Oak, MI 48067, USA; betinahinckel@gmail.com

**Keywords:** biomedical ultrasound, sound diffusivity, frequency-dependent diffusivity, nonlinear acoustics, biological tissue acoustic modelling, COMSOL Multiphysics, nonlinearity parameter B/A, anisotropic tissue properties, viscous attenuation

## Abstract

Ultrasonic wave attenuation in biological tissues arises from complex interactions between mechanical, structural, and fluidic properties, making it essential to identify dominant mechanisms for accurate simulation and device design. This work introduces a novel integration of experimentally measured tissue parameters into time-explicit nonlinear acoustic wave simulations, in which the equations are directly solved in the time domain using an explicit solver. This approach captures the full transient waveform without relying on frequency-domain simplifications, offering a more realistic representation of ultrasound propagation in heterogeneous media. The study estimates both sound diffusivity and viscous damping parameters (dynamic and bulk viscosity) for a broad range of ex vivo tissues (skin, adipose tissue, skeletal muscle, trabecular/cortical bone, liver, myocardium, kidney, tendon, ligament, cartilage, and gray/white brain matter). Four regression models (power law, linear, exponential, logarithmic) were applied to characterize their frequency dependence between 0.5 and 5 MHz. Results show that attenuation is more strongly driven by bulk viscosity than dynamic viscosity, particularly in fluid-rich tissues such as liver and myocardium, where compressional damping dominates. The power-law model consistently provided the best fit for all attenuation metrics, revealing a scale-invariant frequency relationship. Tissues such as cartilage and brain showed weaker viscous responses, suggesting the need for alternative modeling approaches. These findings not only advance fundamental understanding of attenuation mechanisms but also provide validated parameters and modeling strategies to improve predictive accuracy in therapeutic ultrasound planning and the design of non-invasive, tissue-specific acoustic devices.

## 1. Introduction

Ultrasound stands at the forefront of biomedical technologies due to its non-invasive nature and exceptional safety profile [1,2]. This acoustic imaging and therapy modality focuses on being safe, comfortable, efficient, and well tolerated. As such, it offers real benefits in pain relief and function without the burdens or risks of drugs or invasive approaches [3,4]. By utilizing non-audible high-frequency sound rather than ionizing radiation, ultrasound provides real-time visualization of soft tissues, blood flow, and even the developing fetus with minimal risk. Recent advancements allow 3D shear wave elastography [5,6] and high-resolution, real-time imaging of blood flow and microcirculation [7,8]. AI-assisted detection is also being incorporated into diagnostic equipment, enhancing diagnostic speed, accuracy, and image quality during prenatal assessments [9,10]. The inherent low cost and portability, when compared with other imaging technologies, have driven widespread adoption, and as such ultrasound is now among the most commonly used imaging modalities worldwide, with pocket-sized and handheld scanners enabling bedside point-of-care examinations [11]. Besides their established diagnostic roles, ultrasound technologies are also being harnessed for a growing range of therapies. High-intensity focused ultrasound (HIFU) can non-invasively ablate tumors or palliate pain (for example, FDA-approved treatments for fibroids and prostate tissue) [12], while lower-intensity ultrasound waves are being explored to modulate neural circuits and transiently open the blood–brain barrier for targeted drug delivery [13]. Likewise, low-intensity pulsed ultrasound (LIPUS) has been shown to stimulate stem-cell proliferation and accelerate bone and soft-tissue repair [6,14], making it a promising tool for tissue regeneration. Given the limitations in objectively assessing the efficacy of conventional interventions such as orthoses [15,16], ultrasound-based therapies are increasingly being explored as alternative or complementary treatments, particularly in conditions like osteoarthritis [17,18]. Its versatility, safety, and portability highlight ultrasound’s expanding impact across both diagnostic imaging and emerging therapeutic applications.

Poor estimation in biomedical ultrasound computational modeling imposes substantial multi-faceted costs. Clinically, erroneous parameterization in elastography can elevate false-negative rates in tumor detection by 15–30%, delaying critical interventions, while inaccuracies in thermal ablation simulations correlate with 20–25 recurrence rates due to suboptimal dose delivery [19]. Operationally, inefficient algorithms (e.g., conventional 1D cross-correlation requiring >222 s/frame vs. 0.1 s for optimized 2D methods) drastically increase processing latency, energy consumption (by 30–70%), and hardware costs [20,21]. Financially, compensating for estimation errors inflates infrastructure budgets by up to 30% and incurs recurring expenses for model recalibration (e.g., $50 k–$200 k/validation cycle) [22]. Regulatory setbacks from inaccurate models, contributing to 40% of FDA clearance delays for ultrasound devices, further escalate development costs by 1.5–2× [21]. Cumulatively, these factors impede innovation, with flawed in silico trials prolonging R&D timelines by 6–18 months, necessitating robust validation frameworks to mitigate risks.

Modeling ultrasound propagation in biological tissues is inherently challenging, as these media exhibit viscoelasticity, anisotropy, and nonlinear acoustic behavior, which significantly complicate simulation fidelity. For instance, soft tissue viscoelasticity manifests through frequency-dependent dispersion and attenuation of shear and compressional waves, requiring models to account for both elastic and viscous components of the tissue response [23,24]. In recent years, fractional-order viscoelastic models have emerged as a powerful alternative for accurately representing the frequency-dependent attenuation observed in soft tissues. These models, such as the Kelvin–Voigt Fractional Derivative (KVFD) and fractional Zener formulations, provide a physically consistent framework by capturing the cumulative effect of multiple relaxation processes and demonstrating excellent agreement with empirical power-law behavior in both compressional and shear wave propagation [25,26,27,28]. Holm and Näsholm have shown that commonly used fractional wave equations (e.g., Szabo’s, fractional Laplacian) can be derived as low-frequency approximations of more rigorous fractional constitutive models [29]. Experimental comparisons indicate that fractional-calculus-based viscoelastic models often outperform traditional integer-order models in accuracy when describing soft and biological tissues [30,31]. Incorporating fractional-order approaches in the modeling framework of ultrasonic attenuation thus offers an appealing pathway for future enhancements, by allowing better physical interpretability and more accurate parameterization across a broad frequency domain.

On the other hand, anisotropic structures such as muscle and tendon cannot be adequately represented using isotropic acoustic models, since early studies demonstrated that modeling muscle as a transversely isotropic medium provides a much better match to experimental data than isotropic assumptions [32,33]. Beyond speed of sound, attenuation (the energy loss due to viscous and thermal dissipation) is often characterized by a power-law dependency on frequency, yet it remains difficult to integrate accurately into propagative models [24,34]. Furthermore, nonlinearity leads to waveform distortion and harmonic generation, effects not captured by linear acoustic formulations [35,36]. Although attenuation data are commonly measured in ex vivo and phantom tissues, most computational models still rely on simplified, often isotropic and linear representations, which fail to reproduce observed wave behavior in heterogeneous, lossy, or anisotropic tissues [23,37,38]. Multiphysics simulation software like COMSOL Multiphysics is widely employed in ultrasound and cartilage research because it allows precise, coupled finite-element modeling of acoustic, thermal, and mechanical phenomena, which is essential for designing patient-specific therapeutic devices [39,40,41]. Consequently, these advanced simulations require experimentally validated parameters for attenuation and diffusivity along with solver configurations that address dispersion, scattering, and nonlinearity. Without incorporating these complexities, model predictions risk being unreliable for therapeutic and diagnostic applications

Sound diffusivity (*δ*) quantifies how acoustic energy disperses and dissipates within a medium over time and space, effectively linking attenuation and dispersion within a unified framework. Although *δ* appears in models such as the Westervelt or Westervelt–Pennes equations to describe nonlinear thermoacoustic behavior and energy loss mechanisms [42], there is a notable absence of direct experimental measurements of *δ* in biological tissues. Most studies focus on related metrics, such as thermal diffusivity or ultrasonic attenuation coefficients, but do not extract or report the composite parameter of sound diffusivity (as defined in nonlinear wave formulations). For example, El-Brawany et al. measured thermal diffusivity and attenuation in porcine tissues, yet their work did not extend to computing *δ* for use in wave propagation models [43]. This gap becomes crucial because simulation platforms (e.g., COMSOL Multiphysics) often require *δ* to accurately model frequency-dependent losses in nonlinear acoustic media. To the best of our knowledge, no prior experimental studies have quantified sound diffusivity (*δ*) across physiological tissues, making this work a pioneering step toward integrating experimental acoustics with advanced simulation frameworks.

Consequently, many computational models still rely on assumed rather than validated parameters for attenuation and nonlinearity, limiting their predictive accuracy. This underscores the pressing need for an experimental–computational integration strategy: one that not only measures key acoustic properties, such as attenuation, nonlinearity, and diffusivity, but also validates simulation inputs through direct comparison. This work is precisely aimed at closing this gap: by experimentally quantifying tissue parameters and using them in well-configured simulations, we aim to anchor models in physical reality and significantly improve their reliability.

To address critical modeling gaps in biomedical acoustics, this study employs a time-explicit numerical approach, in which the full transient nonlinear acoustic wave equations are integrated in the time domain using an explicit Runge–Kutta solver. This method captures the complete temporal evolution of the acoustic waveform, including arrival times, phase information, and broadband attenuation, without relying on frequency-domain simplifications. The “time-explicit” aspect is experimentally validated by directly comparing simulated and measured time-domain pressure waveforms in multiple tissue types. We experimentally characterize key acoustic and physical properties, namely density, speed of sound (SoS), attenuation coefficient (*α*), and nonlinearity parameter (B/A), across multiple ex vivo tissue types. While density and sound speed are fundamental and commonly reported (e.g., typical soft tissues range from 1430 to 1647 m/s) [44], accurate measurement of attenuation and especially nonlinear parameters like B/A remain less standardized. The B/A parameter, though central to finite-amplitude propagation models, has largely been reported only for soft tissue phantoms or specific organs under limited conditions [45]. By systematically measuring these parameters under controlled hydration and temperature conditions, the present work fills an essential experimental gap. These values are then employed in COMSOL Multiphysics nonlinear acoustics simulations to derive the tissue-specific sound diffusivity *δ*(*f*), which is subsequently validated through rigorous regression against the observed frequency-dependent trends. Ultimately, this integrated experimental–computational approach supports the development of more accurate, physically grounded acoustic simulations of biological media for diagnostic and therapeutic applications.

## 2. Theoretical Background

The propagation of acoustic waves in a medium is classically described by the linear acoustic wave equation, which governs the evolution of pressure fluctuations in response to mechanical perturbations. For a lossless, homogeneous, and isotropic medium, the pressure formulation of the wave equation is given by Equation (1),

(1) $$\nabla^{2}p=\frac{1}{c^{2}}\frac{\partial^{2}p}{\partial t^{2}}$$

where *p* is the acoustic pressure field (Pa), *c* is the speed of sound (SoS) in the medium (m/s), and *t* is time (s). This idealized formulation assumes the absence of dissipative effects and nonlinearities, making it appropriate only for simplified or preliminary analyses.

The SoS *c* is a material-dependent property, influenced by the medium’s elastic and inertial characteristics. In liquids, it is given by Equation (2),

(2) $$c=\sqrt{\frac{K_{s}}{\rho }}$$

where $K_{s}$ is the isentropic bulk modulus (Pa) and ρ is the density of the liquid (kg/m^3^). In solid media, the longitudinal wave speed can be expressed by Equation (3),

(3) $$c=\sqrt{\frac{E(1-\nu )}{\rho (1+\nu )(1-2\nu )}}$$

where E is the Young modulus (Pa) and ν is the Poisson ratio.

In biological tissues, which are composed predominantly of water (typically ~70%), the SoS is not a constant but varies significantly with tissue composition, hydration level, and mechanical properties. Consequently, it is commonly measured experimentally. This spatial heterogeneity in acoustic velocity leads to refraction, scattering, and wavefront distortion, underscoring the importance of accurate spatial modeling in both computational simulations and experimental studies.

While Equation (1) assumes a lossless medium, this simplification does not hold for biological tissues, which exhibit frequency-dependent attenuation. In the literature, acoustic attenuation is often modeled using a power-law formulation, as specified in Equation (4),

(4) $$\alpha \left(f\right)=\alpha_{0}f^{n}$$

where α(f) is the acoustic attenuation coefficient (dB/cm) at frequency f (MHz), $\alpha_{0}$ is the acoustic attenuation coefficient at 1 MHz, and *n* is the frequency exponent, typically ranging between 1 and 2 depending on the tissue type. If this value is close to 1, it means that the acoustic attenuation of the biological tissue is approximately linear.

To account for acoustic attenuation, Equation (1) can be modified by introducing a post hoc correction term that incorporates the frequency-dependent attenuation coefficient α, as shown in Equation (5),

(5) $$\frac{\partial^{2}p}{\partial t^{2}}=c^{2}\nabla^{2}p-2\alpha_{0}c\frac{\partial }{\partial t}{\left(-\nabla^{2}\right)}^{n/2}p$$

where $\alpha_{0}$ and *n* are the same as in Equation (4). The operator ${\left(-\nabla^{2}\right)}^{n/2}$ allows modelling of frequency-dependent loss in the time domain.

COMSOL Multiphysics, however, does now allow the direct implementation of $\alpha \left(f\right)$ in transient/time-explicit domains, only in the frequency domain. As such, we will explore the alternatives for inserting attenuation in the domains that are pertinent to acoustic wave propagation in biological tissues.

Although low-amplitude ultrasound propagation can often be modelled using linear wave theory in other mediums, biological media frequently exhibit nonlinear acoustic behavior even at low amplitudes. As waves propagate, regions of compression travel faster than regions of rarefaction, leading to waveform distortion, harmonic generation, and in extreme cases, the formation of acoustic shocks.

This nonlinear behavior is captured by extending the classical wave equation (Equation (1)) to include a nonlinearity parameter, typically formulated via the Westervelt equation or other second-order approximations, as expressed in Equation (6),

(6) $$\nabla^{2}p-\frac{1}{c_{0}^{2}}\frac{\partial^{2}p}{\partial t^{2}}+\frac{\delta }{c_{0}^{4}}\frac{\partial^{3}p}{\partial t^{3}}=\frac{\beta }{\rho_{0}c_{0}^{4}}\frac{\partial^{2}p^{2}}{\partial t^{2}}$$

where the first two terms form the classic linear wave equation (Equation (1)), the third term introduces frequency-dependent attenuation (from viscosity and thermal conduction), characterized by sound diffusivity δ (m^2^/s), and the right-hand side represents nonlinear propagation, where higher-pressure regions travel faster, leading to wavefront distortion and harmonic generation, scaled by the nonlinearity parameter β [46].

The nonlinearity parameter β is given by Equation (7),

(7) $$\beta =1+\frac{1}{2}\frac{B}{A}$$

where *A* is the linear compressibility, representing how pressure changes with small density fluctuations (approximately linear), and *B* is the nonlinear compressibility, accounting for the pressure build-up under strong compression. The term B/A is often used to denote a nonlinearity parameter given by Equation (7), where $\frac{B}{A}$ is used to calculate β.

It is important to note that the SoS c is replaced by $c_{0}$, which denotes the quiescent (small-signal) sound speed. Since high-pressure regions propagate faster, the actual wave SoS *c* becomes dependent on the local particle velocity *u* (m/s), as described in Equation (8).

(8) $$c=c_{0}+\left(\frac{1}{2}\frac{B}{A}\right)u$$

In COMSOL Multiphysics, the Westervelt equation (Equation (6)) is implemented in a modified form (Equation (9)),

(9) $$\frac{1}{\rho c^{2}}\frac{\partial^{2}p_{t}}{\partial t^{2}}+\nabla \cdot \left(-\frac{1}{\rho }\left(\nabla p_{t}-q_{d}\right)-\frac{\delta }{\rho c^{2}}\frac{\partial p_{t}}{\partial t}\right)=\frac{\beta }{\rho^{2}c^{4}}\frac{\partial^{2}p_{t}^{2}}{\partial t^{2}}+Q_{m}$$

where $p_{t}$ is the total pressure (Pa), and $q_{d}$ and $Q_{m}$ are dipole and monopole sources, respectively, that might be inserted in the medium.

Although both Equations (6) and (9) model attenuation through δ, neither incorporates the frequency-dependent attenuation $\alpha \left(f\right)$ as described in Equation (4), which is the parameter most commonly reported in the literature. COMSOL does not directly prescribe $\alpha \left(f\right)$ in time-explicit formulations, creating a disconnect between measured attenuation data and input parameters, and as such direct δ measurement can aid in accurate modelling of nonlinear problems.

This highlights a critical gap in the literature: the lack of direct measurements or universally accepted methods for converting $\alpha \left(f\right)$ into an accurate sound diffusivity δ. Addressing this gap is essential for improving the predictive accuracy of nonlinear acoustic simulations in biological tissues.

Therefore, this investigation aims to establish a straightforward experimental approach to characterize acoustic wave propagation in biological tissues and to demonstrate how the resulting data can be integrated into COMSOL Multiphysics to derive the necessary parameters and enable accurate simulation of acoustic wave behavior.

## 3. Materials and Methods

This study was divided into two main components: experimental characterization and computational modeling. In the experimental phase, key acoustic parameters (density ρ, SoS c, α(f), and the nonlinearity parameter B/A) were measured to evaluate the suitability of the chosen methodology and to compare the results with values reported in the literature. In the computational phase, a regression analysis was performed to assess how well the attenuation model parameters fit the experimental data.

### 3.1. Experimental Characterization

The biological samples used in this study were acquired fresh and tested immediately to minimize any post mortem degradation. All measurements were conducted under controlled temperature and humidity conditions (22 °C, relative humidity 60%), and the samples were kept fully hydrated throughout the process. When immediate testing was not possible, the samples were stored in an appropriate physiological saline solution (phosphate-buffered saline) at 4 °C, ensuring the preservation of tissue integrity, hydration, and acoustic properties. Table 1 summarizes the number of samples, their biological origins, and the types of tissues analyzed.

**Figure 1:**
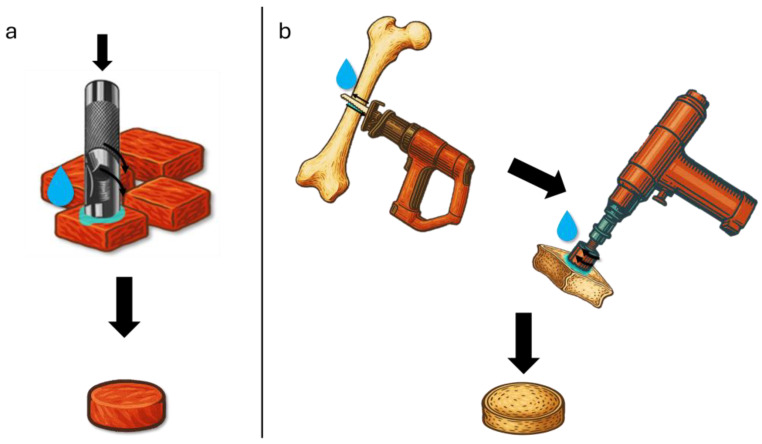
Illustration of the sample preparation process—(**a**) Soft biological tissues were cut into 24 mm discs using a handheld hollow punch, rotated through the sample in a single motion to minimize shear distortion and edge deformation. (**b**) Bone samples were first trimmed into manageable blocks using a low-speed linear reciprocating saw under continuous saline irrigation to reduce thermal and mechanical damage, then the resulting bone blocks were shaped into cylindrical specimens using a 24 mm hole saw drill bit (without a guide tip), operated at low speed and under coolant, to achieve final dimensions suitable for acoustic testing while preserving structural integrity.

Circular soft tissue specimens (24 mm diameter, ~2 mm thick) were prepared using a handheld sterile hollow punch, rotated 90° in a single motion to minimize shear and ensure smooth edges (Figure 1a), as was previously performed in other studies [47,48]. To further reduce artifact, tissue was kept at 4 °C and blades were changed after every five cuts. Bone cylinders were obtained by first trimming blocks with a low-speed diamond saw under continuous saline irrigation to limit thermal damage, as was previously reported [49,50], then using a 24 mm hole-saw drill bit (no guide tip) at low RPM with coolant to produce cylindrical samples; both techniques conform to pathology-grade sample preparation protocols (Figure 1b).

To determine the density of each sample, the radius and thickness were measured using a high-precision digital caliper to calculate volume, while the mass was obtained using a micrometric precision balance.

The SoS and attenuation coefficient were calculated by adapting the methodology previously described by Fernandes et al. for characterizing elastomeric composites used in medical phantoms [51]. However, certain soft biological tissues, such as brain, liver, kidney, and cartilage, exhibited extreme deformability, making conventional measurement techniques unsuitable due to sample compression.

**Figure 2:**
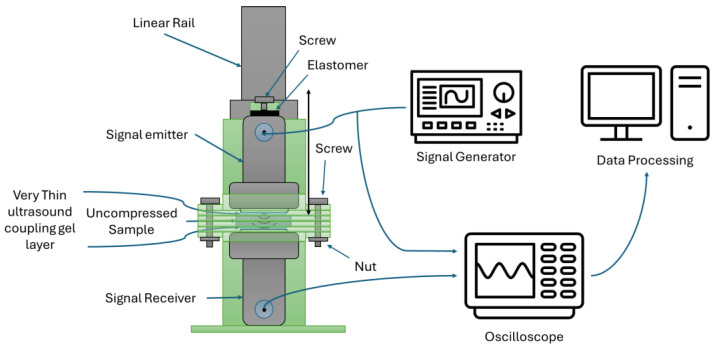
Schematic of the experimental setup used for ultrasonic wave propagation through biological samples. The sample is positioned between two aligned Mitech transducers, separated by 1 mm spacer rings to ensure constant sample thickness. An elastomeric layer is placed above the upper transducer, allowing the securing screw to apply pressure without causing transducer deformation. The system is excited by a function generator and monitored with an oscilloscope to capture time-of-flight and frequency-domain responses. The green pieces represent a custom-made 3d print support for the sample and transducers.

To address this, the experimental setup was modified based on the principle of an impedance tube (Kundt’s tube). Specifically, the tissue samples were placed between two ultrasound transducers, separated by spacer rings with a fixed thickness of 1 mm. This ensured that the sample height remained constant and was equivalent to the combined height of the spacers, allowing for consistent and non-compressive measurement of acoustic wave propagation (see Figure 2).

Acoustic signals were propagated using Mitech ultrasonic (China) transducers equipped with 24 mm diameter PZT discs, operating at frequencies of 0.5, 1, 2, 4, and 5 MHz. Two distinct signal amplitudes were employed at each frequency to extract different acoustic parameters.

For measuring the SoS c and the acoustic attenuation coefficient α(f), a low-amplitude signal (10 V) was used. The SoS was characterized by Equation (10),

(10) $$c=\frac{l}{t}$$

where *l* is the sample thickness (m) and *t* is the time of flight between the transmitting and receiving transducers (s).

The acoustic attenuation coefficient $\alpha \left(f\right)$, in dB/cm, was determined using Equation (11),

(11) $$\alpha (f)=\frac{20{log}_{10}\left(\frac{A_{1}}{A_{2}}\right)}{l}$$

where $A_{1}$ is the amplitude of the reference signal (baseline, no sample) and $A_{2}$ is the amplitude of the transmitted signal through the sample.

**Figure 3:**
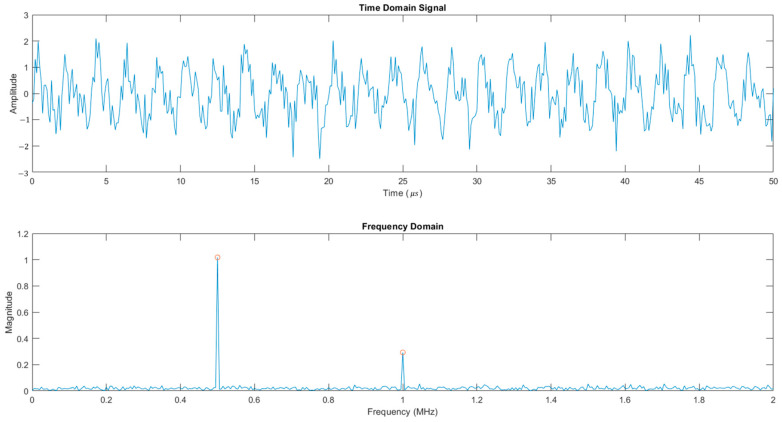
Time and frequency domain representation of an ultrasonic signal used to calculate the nonlinearity parameter $\frac{B}{A}$. The fundamental component at 0.5 MHz and the second harmonic at 1 MHz are highlighted in the frequency spectrum. The circle around the peaks represents the maximum at each of the frequencies.

To measure the B/A nonlinearity parameter, a high-amplitude signal (100 V) was used. The parameter was computed using Equation (12),

(12) $$\frac{B}{A}=\frac{8\rho_{0}c_{0}^{3}}{{\left(2\pi f\right)}^{2}}\frac{A_{2}^{measured}}{A_{1}^{2}}10^{\frac{\left(\alpha \left(2f\right)-2\alpha (f)\right)l}{20}}$$

where $\rho_{0}$ is the density of the sample (kg/m^3^), $c_{0}$ is the SoS (m/s), f if the fundamental frequency (MHz), $A_{1}^{2}$ is the amplitude of the fundamental frequency in the spectrum, and $A_{2}^{measured}$ is the amplitude of the second harmonic in the spectrum as exemplified in Figure 3.

A correction factor was included to compensate for the fact that attenuation is frequency-dependent, and thus the second harmonic undergoes greater attenuation than the fundamental. This adjustment ensures a more accurate estimation of the nonlinearity parameter.

The experimentally obtained values will be compared with existing literature to validate the measurement methodology and will also serve as inputs for the computational modeling phase, particularly for the estimation of the sound diffusivity δ.

### 3.2. Simulation Setup (COMSOL Multiphysics)

To calculate the sound diffusivity in COMSOL Multiphysics (version 5.3), the Nonlinear Pressure Acoustics, Time Explicit (NATE) package was employed. This module is based on the discontinuous Galerkin method and utilizes a time-explicit solver suitable for modeling nonlinear acoustic wave propagation.

The input signal applied to the biological samples was replicated in the simulation environment. To ensure sufficient frequency resolution, the FFT of the input signal was analyzed to identify its maximum frequency content, which was then doubled to define the maximum frequency range resolved by the simulation. This approach ensured that both fundamental and harmonic components relevant to nonlinearity were accurately captured.

**Figure 4:**
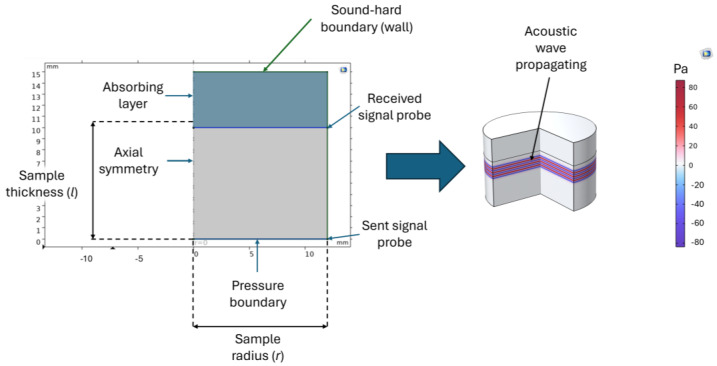
Schematic of the 2D axisymmetric geometry used for simulating acoustic wave propagation in COMSOL Multiphysics. The model includes a biological sample domain (gray), an absorbing layer (blue) to eliminate reflected waves, and defined boundaries for the pressure input, signal reception, and wall constraints. Axial symmetry (r = 0) is applied along the left edge to reduce computational complexity while maintaining physical accuracy.

The experimentally measured average density, SoS, and B/A nonlinearity parameter were used as input parameters for the simulation. The geometry was constructed based on the average radius *r* and thickness *l* of the tested biological samples and is illustrated in Figure 4. To reduce the number of degrees of freedom and computational cost, a 2D axisymmetric domain was employed. The input signal was applied as a pressure boundary condition, using the experimentally acquired waveform defined as a time-dependent function. Two probes were positioned within the geometry: one near the source to monitor the emitted signal, and another at the distal end of the sample to capture the received waveform. A sound-hard (wall) boundary condition was assigned to the remaining external edges of the domain. To minimize artificial reflections and ensure accurate simulation of wave propagation, an absorbing layer was included at the far end of the model.

The mesh was designed to satisfy a Courant–Friedrichs–Lewy (CFL) condition of 0.2, ensuring numerical stability for most biological tissues. However, for materials exhibiting high acoustic attenuation, such as trabecular and cortical bone, a denser mesh was required to accurately capture wave behavior. In these cases, the number of elements per wavelength was doubled, yielding a more stringent CFL condition of 0.1.

The element size was computed using Equation (13):

(13) $$\Delta x=\frac{c\Delta }{f\cdot CFL}$$

where c is the SoS in the desired sample (m/s), CFL is the desired CFL condition, f is the maximum frequency (Hz), and Δx is the maximum element size (m). Triangular elements were employed throughout the mesh. The resulting domain elements per sample configuration are listed in Table 2.

Time stepping was carried out using the Runge–Kutta method (RK34) with a fixed time step to ensure numerical stability. The simulation duration was carefully adjusted to match the time required for the input signal to fully propagate through the sample, thereby avoiding unnecessary computations beyond the wave’s transit period.

The MUMPS solver (Multifrontal Massively Parallel Sparse direct solver) was used with a memory allocation factor of 1.2 to ensure adequate memory resources for handling large sparse matrices while avoiding overconsumption that could lead to instability or excessive computational load. This allocation strikes a balance between solver performance and memory efficiency, particularly important for simulations involving fine meshes and high-frequency wave propagation in heterogeneous media.

Two dissipative approaches were explored in COMSOL Multiphysics: one by directly solving for the sound diffusivity δ using the nonlinear wave equation (Equation (6)), and another by employing the viscous fluid model, where the diffusivity is derived from physical fluid properties as shown in Equation (14),

(14) $$\delta =\frac{1}{\rho }\left(\frac{4}{3}\mu +\mu_{B}\right)$$

where the new term μ is the dynamic viscosity (Pa·s), $\mu_{B}$ is the bulk viscosity (Pa·s), and ρ is the density of the medium (kg/m^3^). This equation is based on previously described methods in the literature, in which damping due to thermal loses are ignored [52,53]. Although this viscosity-based method is computationally more demanding, it provides a more physically grounded model of attenuation by incorporating shear and compressive dissipation effects. This dual approach allows both empirical fitting and physics-informed modeling of acoustic energy loss in biological tissues.

The experimentally acquired signal was used as the objective function for parameter estimation, allowing the simulation to be iteratively adjusted until the numerically generated signal closely matched the experimental response in the time domain.

The BOBYQA algorithm was employed in conjunction with the least-squares parameter estimation method, using an optimality tolerance of 0.1. The initial parameter bounds and tolerances are presented in Table 3. In cases where the optimization reached a boundary limit, the corresponding maximum value was doubled to allow for further exploration of the solution space.

The problem was then formulated in COMSOL Multiphysics as a least-squares problem. The general form of a least-squares optimization problem is expressed as Equation (15),

(15) $${min}_{\theta }\;J\left(\theta \right)=\sum_{i=1}^{N}{\left[y_{i}^{model}\left(\theta \right)-y_{i}^{observed}\right]}^{2}$$

where θ is a vector of unknown parameters to be estimated, $y_{i}^{observed}$ represents the observed data points, $y_{i}^{model}\left(\theta \right)$ represents the predicted data points from the model using parameters θ, *N* is the total number of data points, and $J\left(\theta \right)$ is the cost loss function to be minimized. Applying this formulation to Equation (9), Equation (16) is formed:

(16) $${min}_{\delta }\;J\left(\delta \right)=\sum_{i,j}{\left[p^{simulation}\left(x_{i},t_{j};\delta \right)-p^{observed}(x_{i},t_{j})\right]}^{2}$$

The simulation results, derived from validated input data and calibrated using parameter estimation techniques, provide a reliable foundation for evaluating acoustic wave propagation in biological tissues and for extracting meaningful physical parameters such as sound diffusivity. This integrated approach enables direct comparison between experimental and simulated data, strengthening the overall validity of the proposed methodology.

### 3.3. Post-Processing

All data analysis and post-processing were conducted using MATLAB R2024b. For the characterization of acoustic wave propagation, signals acquired using a 10 V input were first processed with a second-order Butterworth low-pass filter. The cutoff frequency was set to twice the center frequency of the signal to eliminate high-frequency noise while preserving relevant harmonic content.

For the nonlinearity analysis using a 100 V excitation signal, a second-order Butterworth low-pass filter was also applied, but with a cutoff frequency set to four times the center frequency. FFT analysis was performed using a Hanning window with zero-padding to increase spectral resolution. This ensured that the second harmonic remained intact while higher-order noise components were suppressed, enabling accurate identification of nonlinear effects. The peak amplitudes for fundamental and second harmonic were extracted using local maxima detection within ±10 kHz around the expected frequency.

To calculate the frequency-dependent attenuation coefficient $\alpha \left(f\right)$, a power-law model was applied. For computational efficiency, Equation (4) was linearized into the logarithmic form shown in Equation (17):

(17) $${log}_{10}\left(\alpha \left(f\right)\right)={log}_{10}\left(\alpha_{0}\right)+n\cdot {log}_{10}\left(f\right)$$

This transformation enabled the use of MATLAB’s *fitlm* function to perform linear regression and extract the attenuation coefficient $\alpha_{0}$ and exponent n.

For the analysis of simulated parameters, multiple regression models (linear, exponential, power law, and logarithmic) were tested. This allowed for the prediction of parameter behavior across intermediate frequencies, facilitating interpolation and aiding the validation of both experimental and computational results. *R*^2^ values were evaluated for all regressions to ensure model validity, using Equation (18),

(18) $$R^{2}=1-\frac{\sum_{i}{\left(y_{i}-\hat{y_{i}}\right)}^{2}}{\sum_{i}{\left(y_{i}-\bar{y}\right)}^{2}}$$

where $y_{i}$ is the experimental value, $\bar{y}$ is the average value of *y*, and $\hat{y_{i}}$ is the predicted value for *i*.

Custom MATLAB scripts were developed to automate batch filtering, FFT computation, and regression fitting.

Together, these post-processing steps ensured consistent and reliable extraction of acoustic parameters across all samples. By combining advanced signal filtering, frequency-domain analysis, and robust regression techniques, the methodology enabled precise characterization of both linear and nonlinear acoustic behaviors, forming a solid foundation for subsequent computational validation and model calibration.

## 4. Results and Discussion

Section 4 is divided into two main parts. The first focuses on the experimental findings, where the validity and reliability of the measurement methodology are assessed. Key acoustic and physical parameters are quantified and compared with values reported in the literature to ensure experimental consistency. These validated results are then used as foundational inputs for the second part, which presents the computational analysis. There, experimentally derived parameters are employed in numerical simulations to estimate sound diffusivity and assess their suitability for accurately modeling wave propagation in biological tissues.

### 4.1. Experimental Results

This section explores the experimental results related to density, speed of sound, acoustic attenuation coefficient, and the B/A nonlinearity parameter, which will serve as inputs for computational modeling. To improve readability, additional experimental data have been omitted from the main text and is included in Appendix A.

#### 4.1.1. Density

**Figure 5:**
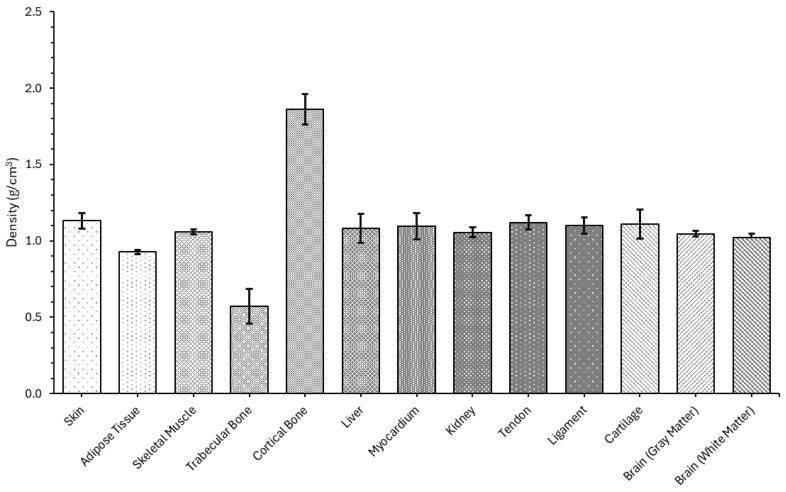
Measured density (g/cm^3^) of various biological tissues, showing mean values with standard deviation.

A bar chart that illustrates the experimentally measured density values for various biological tissues, highlighting both soft and hard tissues, can be seen in Figure 5. A table containing all the experimental values is present in Table A1.

Among all samples, cortical bone exhibited the highest density (1.86 ± 0.10) g/cm^3^, consistent with its mineralized structure [54]. Conversely, trabecular bone had the lowest density (0.57 ± 0.12) g/cm^3^, reflecting its porous architecture [55].

Soft tissues, including skin (1.13 ± 0.05) g/cm^3^, tendon (1.12 ± 0.05) g/cm^3^, and ligament (1.10 ± 0.05) g/cm^3^, displayed slightly higher densities compared to organ tissues such as liver (1.08 ± 0.10) g/cm^3^, kidney (1.06 ± 0.03) g/cm^3^, and brain. Notably, adipose tissue showed the lowest density among soft tissues (0.93 ± 0.02) g/cm^3^, aligning with its high lipid content and low water fraction. Both gray matter (1.05 ± 0.02) g/cm^3^ and white matter (1.02 ± 0.02) g/cm^3^ of the brain were slightly less dense than muscle, liver, and connective tissues, a result attributed to their cellular composition and myelin content.

The relatively low standard deviation values for most tissues indicate consistency in sample preparation and measurement technique. However, the higher variation in trabecular and cortical bone likely stems from microstructural heterogeneity and differences in marrow content.

The measured ex vivo hydrated tissue density measurements were compared with recent literature values for human, porcine, and bovine tissues. In general, our measured values agree closely with reported densities for hydrated tissues. For example, the skin density we measured (~1.07 g/cm^3^) is slightly below the ICRP composition value of 1.10 g/cm^3^ [56]. Adipose tissue (fat) is well known to be low-density and the measured ~0.92 g/cm^3^ is near the reported value of 0.9 g/cm^3^ [57]. Similarly, skeletal muscle density (~1.05 g/cm^3^) matches the ≈1.06 g/cm^3^ often cited [58,59]. Cortical bone is very dense and the measured ~1.93 g/cm^3^ agrees with the ~1.9–2.0 g/cm^3^ range reported for human bone [60]. Trabecular bone has a much lower apparent density due to high porosity, with the literature reporting vertebral wet densities ~0.09–0.35 g/cm^3^ [61], which is lower than the measured samples ~0.72 g/cm^3^ (suggesting the measured sample was relatively dense trabecular or contained marrow). Measured liver density (~1.06 g/cm^3^) is consistent with porcine studies (≈1.07 g/cm^3^) [62]. The myocardium (heart muscle) density (~1.07 g/cm^3^) falls in the range of ~1.05–1.09 g/cm^3^ reported (human often taken as 1.055 g/cm^3^ [63]; rat measured ~1.09 g/cm^3^ [64]). Kidney density (~1.08 g/cm^3^) also matches the ~1.04–1.05 g/cm^3^ reported in ICRP and imaging literature [65]. Tendon and ligament (collagenous connective tissues) are quite dense with the measured values (~1.22 and 1.19 g/cm^3^) being close to 1.12 g/cm^3^ found for tendon in the literature [66] (ligament densities are similar but not well tabulated). Cartilage (~1.17 g/cm^3^) aligns with the literature (~1.15 g/cm^3^) [67]. Finally, brain gray and white matter densities (~1.04 and 1.02 g/cm^3^) are somewhat below the ~1.08 (gray) and ~1.04 (white) g/cm^3^ reported for human brain [68], possibly reflecting hydration state differences. Overall, each measured value is within ~5–10% of literature norms, indicating good consistency.

These density values are fundamental inputs for acoustic modelling, influencing impedance, wave speed, and attenuation in simulations. The consistency of most results with reported literature supports the validity of the adopted methods and reinforces the suitability of the samples for further acoustic analysis in COMSOL Multiphysics.

#### 4.1.2. Speed of Sound

**Figure 6:**
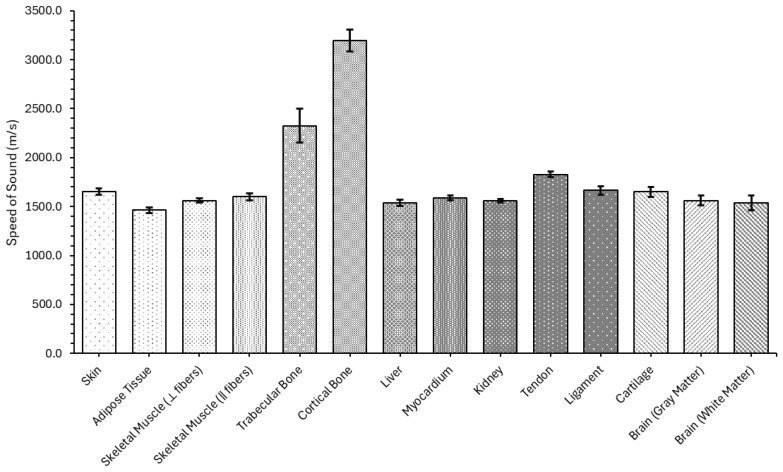
Speed of sound (m/s) in different biological tissues and respective standard deviations.

The measured speed of sound across the evaluated tissues reveals a clear and physiologically consistent trend, as exhibited in Figure 6 and more clearly specified in Table A2.

As expected, cortical bone exhibited the highest SoS, with an average value of (3193.95 ± 113.28) m/s, followed by trabecular bone at (2325.38 ± 172.80) m/s. These values reflect the increased stiffness and density of osseous tissues, with trabecular bone showing greater variability due to its porosity and microstructural heterogeneity. Among soft tissues, the SoS clustered between approximately 1460 and 1660 m/s. Skin (1654.93 ± 32.82) m/s, tendon (1827.43 ± 29.43) m/s, and ligament (1663.68 ± 42.29) m/s registered higher values compared to adipose tissue (1461.77 ± 26.11) m/s, consistent with their more fibrous or collagen-rich composition. Skeletal muscle showed directional dependence, with faster propagation along the fibers (1598.68 ± 34.34) m/s than across them (1562.18 ± 20.76) m/s, confirming its anisotropic behavior. Visceral organs, including the liver (1539.69 ± 33.06) m/s, kidney (1556.87 ± 17.38) m/s, and myocardium (1585.83 ± 24.16) m/s, demonstrated tightly grouped values, consistent with their cellular structure and high water content. Finally, cartilage (1650.80 ± 51.95) m/s presented slightly elevated values, as expected due to its semi-rigid matrix, while brain tissues exhibited a lower SoS, with gray matter (1562.09 ± 53.55) m/s and white matter (1538.46 ± 76.33) m/s falling within typical ranges.

In most soft tissues, recent ex vivo measurements closely match classic literature values. Human skin had a value of ~1655 m/, agreeing with the reported values of 645 m/s in epidermis and 1595 m/s in dermis [69]. Adipose fat measured ≈1460 m/s, also similar to reported values (~1450 m/s) [70] although slightly higher. The measured values of skeletal muscle (calf) data suggest ~1600 m/s parallel to fibers and ≈1560 m/s perpendicular, which is comparable to standard references that disregard anisotropy and report a median SoS in the ~1430–1579 m/s range [70]. Bone shows the largest variation. Cortical bone is very stiff (~3200 m/s average), which is consistent with the literature that reports an average value of ~3400 m/s, while trabecular (spongy) bone ranges widely (~2325 m/s) but is in accordance with the literature (≈1500–2900 m/s) depending on density [71]. Liver speed measured ~1540 m/s, close to the commonly used 1550 m/s of a healthy human [72]. Heart muscle (myocardium) was ~1585 m/s, slightly higher than the nominal 1576 m/s reported in the literature [73]. Tendons and ligaments (dense collagen) obtained a value of ~1830 m/s and ~1660 m/s, which are reported to be high-speed (~1600–1700 m/s) [71], although there is a lack of reported values specifically for ligament. Cartilage in bovine joints measured a value of 1650 m/s, which is in slightly higher than the previously published~1627 m/s [74], with a lack of reported values for human subjects. Brain tissue speeds were slightly higher (~1560 m/s and ~1540 m/s for gray and white matter, respectively) than previously reported values (~1550 m/s and ~1540 m/s for gray and white matter, respectively [75]) with no large anisotropy reported.

Overall, the measured values align well with literature expectations and reflect appropriate experimental control across tissue types. Experimental values from recent human and animal studies generally confirm the obtained values: most deviations are under ~5%. The largest spread occurs in heterogeneous tissues (bone) and where anisotropy matters (muscle fibers). No major anomalies were observed. These speed of sound values were used as input parameters for acoustic wave propagation simulations in COMSOL Multiphysics.

#### 4.1.3. Acoustic Attenuation Coefficient

**Figure 7:**
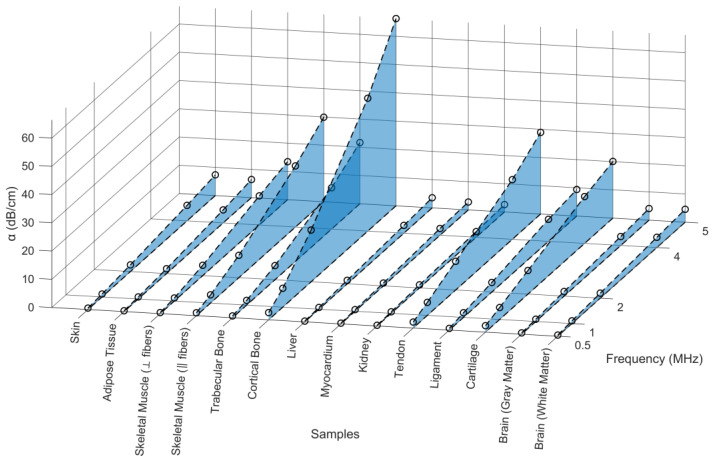
Frequency-dependent ultrasound attenuation coefficient (α, dB/cm) for various biological tissues.

The measured speed of sound across the evaluated tissues reveals a clear and physiologically consistent trend, as exhibited in Figure 7 and Table 4, and the values more clearly specified in Table A2.

The measured α across the evaluated tissues was well described by the power-law model (Equation (4)). The power law fits showed extremely high coefficients of determination ($r^{2}>0.99$) for most tissues, confirming that the experimental data adhered closely to a predictable frequency-dependent behavior.

Soft tissues such as skin (Figure A1A), adipose tissue (Figure A1B), liver (Figure A2A), myocardium (Figure A2B), kidney (Figure A2C), ligament (Figure A3A), and brain gray and white matter (Figure A3C) exhibited relatively low $\alpha_{0}$ values (typically < 1.5 dB/cm) and frequency exponents ranging from approximately 1.0 to 1.3. These results are consistent with the literature and reflect their highly hydrated, viscoelastic nature, where attenuation increases nearly linearly with frequency. Skeletal muscle (Figure A1C) presented a marked anisotropy in attenuation behavior. When waves propagated parallel to muscle fibers, both $\alpha_{0}$ and *n* were substantially higher ($\alpha_{0}$ = 2.87 dB/cm, *n* = 1.46) compared to the perpendicular orientation ($\alpha_{0}$ = 1.15 dB/cm, *n* = 1.56). The elevated *n* values in both directions indicate a more complex viscoelastic response, and the difference between orientations underscores the importance of structural anisotropy in biological media. Cortical bone (Figure A1D), tendon (Figure A2D), and cartilage (Figure A3B) displayed some of the highest attenuation coefficients. Cartilage (Figure A3B) demonstrated a moderate frequency dependence (*n* = 0.96) but a high $\alpha_{0}$ = 4.34 dB/cm, consistent with its semi-rigid matrix and compressive load-bearing properties. Cortical bone (Figure A1D) and tendon (Figure A2D), in contrast, had both high $\alpha_{0}$ (4.62 and 6.78 dB/cm, respectively) and relatively strong frequency dependence (*n* = 1.12 and 1.41, respectively), suggesting that attenuation is strongly influenced by their fibrous, dense structures and collagen alignment. Trabecular bone (Figure A1D), though less dense than cortical bone, also exhibited significant attenuation ($\alpha_{0}$ = 1.78 dB/cm, *n* = 1.52) and high standard deviations due to its porous and heterogeneous nature. Brain grey and white matter (Figure A3C) demonstrated the lowest frequency dependence (*n* ≈ 0.96–1.03), which is in line with their highly aqueous content and lack of fibrous reinforcement [76]. The small differences in $\alpha_{0}$ between gray and white matter likely arise from microstructural differences, particularly due to lipid-rich myelination in white matter [77].

The high $R^{2}$ values (>0.996 in nearly all cases) affirm that the fitted parameters are reliable and representative of the physical behavior of each tissue type. The slightly lower $r^{2}$ values observed in myocardium and ligament (0.9968 and 0.9960, respectively) may reflect biological variability or measurement noise but remain highly acceptable for modelling purposes.

Comparing with the literature, most soft tissues follow a nearly linear frequency law (*n* ≈ 1) with *α*_0_ of order 0.5–1 dB/cm [78,79]. For example, our own muscle and kidney data fall within these ranges. Notably, skeletal muscle is anisotropic and has been reported in the literature that attenuation ⟂ to fibers is lower (~1.1 dB/cm/MHz) than ‖ to fibers (~2.9 dB/cm/MHz) [80], comparable to the measured results (⟂~1.150 dB/cm and ‖~2.870 dB/cm). Myocardium shows a similar anisotropy, giving rise to a large deviation (~1.0–1.8 dB/cm/MHz) [79,81], similar to the measured values. Brain tissue has low attenuation; published white-matter slopes are ≈0.94 dB/cm/MHz, while gray matter is slightly lower [82], once again in accordance with the measured results. By contrast, connective tissues attenuate more strongly. Tendon and ligament (rich in collagen [83]) have *α*_0_ ~4–5 dB/cm/MHz, several times higher than typical soft organs reported in the literature [78,79]. Cartilage has been reported to be relatively attenuating (*α*_0_ ≈ 4.0 dB/cm/MHz) [84], in accordance with our results. Bone is most extreme: published broadband slopes for cancellous (trabecular) bone reach 1–61 of dB/cm/MHz, while cortical bone is ~5–12 dB/cm/MHz [85], agreeing with the obtained results. These values greatly exceed the FDA reference of 0.3 dB/cm/MHz and reflect strong scattering/absorption in mineralized tissue [86].

Overall, our measured trends agree well with the literature. Soft tissues (skin, fat, liver, kidney, brain) cluster around *α*_0_ ≈ 0.5–1.0, *n* ≈ 1, whereas collagenous tissues (tendon, ligament, cartilage) and bone show much higher *α*_0_ [80,85]. Deviations arise from anisotropy (muscle, heart) and heterogeneity (trabecular porosity), but no large systematic discrepancies were noted between our measurements and published values.

#### 4.1.4. B/A Nonlinearity Coefficient

**Figure 8:**
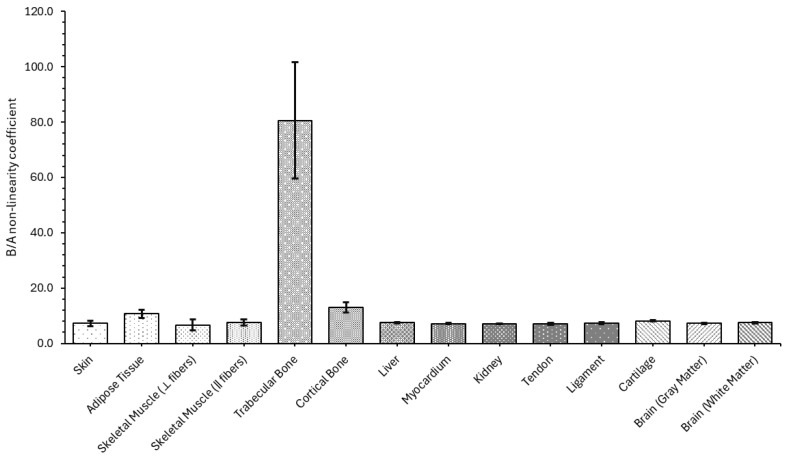
Nonlinearity parameter (B/A) for different biological tissues.

The measured B/A nonlinearity parameter values reveal expected trends across tissue types, reflecting their mechanical and acoustic properties, as demonstrated in Figure 8 and more clearly in Table A4.

Soft tissues such as skin, liver, kidney, tendon, ligament, and brain all cluster around B/A values between 7.0 and 7.5, with low standard deviations, indicating consistent and repeatable measurements. Adipose tissue stands out with a higher B/A of (10.75 ± 1.46), likely due to its compressible, lipid-rich structure, which enhances nonlinear response. Similarly, cartilage and skeletal muscle (‖ fibers) show slightly elevated values, at 8.12 and 7.61, respectively, consistent with their fibrous composition and anisotropic mechanical response. In contrast, trabecular bone presents a markedly higher B/A value of (80.58 ± 21.03), which aligns with expectations given its porous architecture and strong nonlinear elastic response under acoustic loading. Cortical bone, while also more nonlinear than soft tissues, has a lower B/A of (12.95 ± 1.90), reflecting its denser and more homogeneous structure. The significantly higher standard deviation in trabecular bone reflects its inherent microstructural variability.

Soft tissues (skin, muscle, liver, myocardium, kidney, tendon, ligament, gray brain) all measure a B/A nonlinearity parameter of ≈6.5–7.5. This matches literature reports of B/A ≈ 7 for non-fatty soft organs, which indicates that soft tissues must have a value of approximately 7 [87,88]. Adipose tissue (fat) was much higher (10.75) when compared with soft tissues but this is consistent with prior data, where it has been reported that various body fats have B/A ≈ 9.6–10.8 [45] and ≈11 (highest among soft tissues) [88]. Our findings observed skeletal muscle B/A‖ (7.61) > B/A⊥ (6.63), contrary to the literature, which usually reports an isotropic value ~7 [88], but muscle is mechanically anisotropic [51,89]. The higher value along fibers may arise from structural alignment, although published B/A values rarely distinguish orientation. The deviation is modest (within literature range) and plausibly due to fiber orientation. The measured trabecular bone (80.6) and cortical bone (12.95) far exceed values for soft tissue. No standard literature B/A values for bone were found, but this is reasonable: bone’s very high elastic modulus [60,90] and hierarchical microstructure [55] produce extreme nonlinearity. The trabecular result (with large SD) likely reflects its porous, marrow-filled nature [54]. Although our trabecular B/A is exceptionally large, it is qualitatively consistent with the expectation that rigid bone has much higher B/A than soft tissue. Measured cartilage yielded B/A ≈ 8.12, somewhat above the ~7 of soft tissue, consistent with its stiff collagen–proteoglycan matrix [91]. No specific prior B/A data measurement in the literature was found. Tendon and ligament (which also have dense collagen [92]) were near 7, similar to muscle, perhaps because they lack large lipid content [93]. Reported literature states that B/A tends to increase with specimen structural hierarchy, which may explain cartilage’s elevated nonlinearity [88]. White brain matter (myelin-rich) also showed a slightly higher B/A (7.48) than gray (7.22), again reflecting extra lipid content in white matter [94,95]. This aligns qualitatively with the trend that fat/lipid increases acoustic nonlinearity [88].

Overall, the distribution of B/A values corresponds well to tissue type and structure, validating the experimental setup and reinforcing the importance of nonlinear parameters for modeling high-intensity acoustic propagation in biological media. These parameters are essential for accurately simulating wave distortion, harmonic generation, and energy deposition in nonlinear acoustic fields, particularly in therapeutic ultrasound applications.

### 4.2. Computational Results

To investigate the frequency-dependent attenuation behavior of biological tissues, a series of computational simulations were performed to estimate sound diffusivity, dynamic viscosity, and bulk viscosity across a representative range of soft and hard tissues. For the sake of comparison, a typically adopted linear solver was also employed alongside our proposed approaches, enabling direct benchmarking of performance and accuracy, using the viscous attenuation coefficients derived in this work. These results provide insight into the dominant dissipative mechanisms and inform the selection of appropriate regression models for acoustic parameterization.

#### 4.2.1. Acoustic Wave Propagation (0.5 Megahertz)

**Figure 9:**
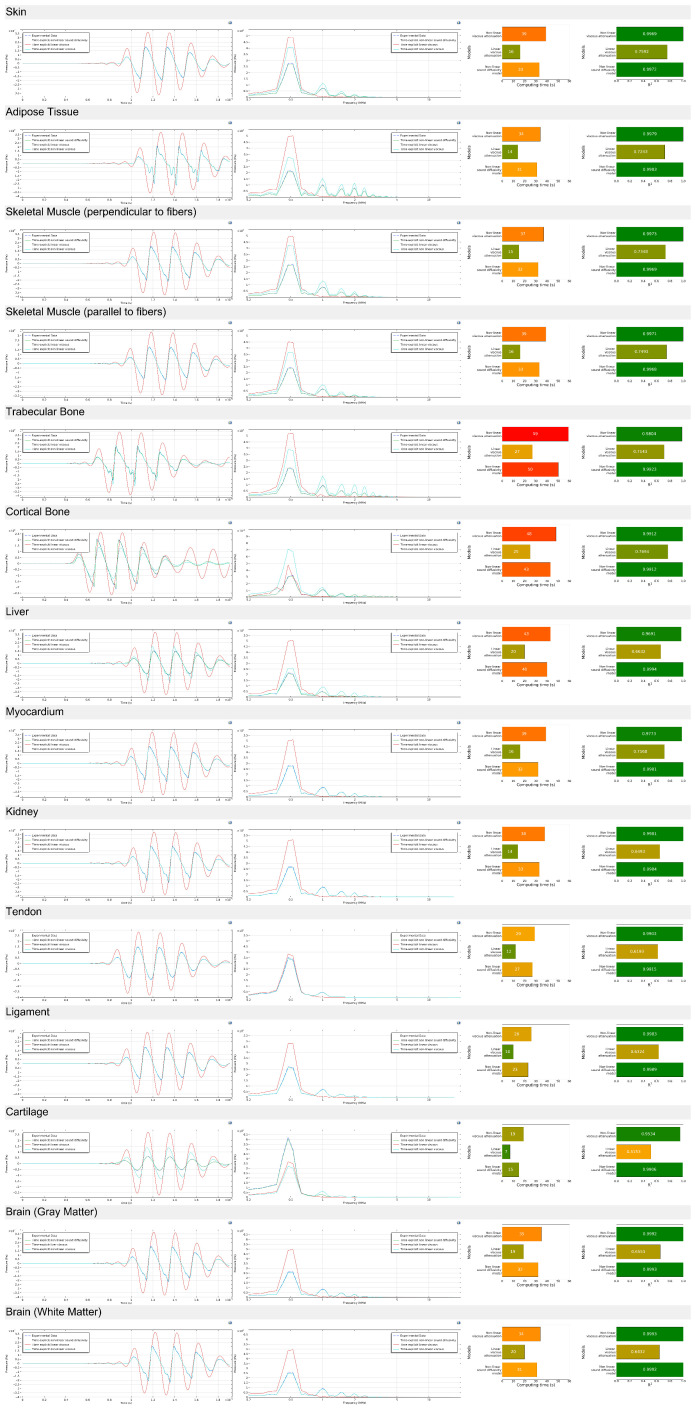
Time domain, frequency domain, computing time, and coefficient of determination (R^2^) of the studied samples at 0.5 MHz.

To assess the accuracy and robustness of the proposed models, the simulated ultrasound waveforms were compared against experimental measurements in both the time and frequency domains for all investigated tissue types. Figure 9 presents a comprehensive overview of this validation process, illustrating results for all samples. For each tissue, the plots display the temporal waveforms, corresponding frequency spectra, computation time, and coefficient of determination (R^2^) values for the three modeling approaches: the linear solver, the sound diffusivity model, and the viscous attenuation model.

The time-domain results demonstrate that across nearly all tissues, the linear solver overestimates the amplitude decay and underestimates the arrival time of secondary wave cycles, resulting in visibly poorer waveform alignment with experimental data. In contrast, both the sound diffusivity and viscous attenuation models capture the waveform envelope and phase more accurately, with the diffusivity model consistently producing the closest match to the measured signal, particularly in tissues with higher attenuation such as cortical bone, tendon, and cartilage. However, the nonlinear solvers also introduced some instability in the time-domain signal for certain tissues, most notably cortical bone, liver, and cartilage, where small oscillatory artifacts appeared due to the combination of high attenuation and numerical stiffness in the solver.

In the frequency domain, the superiority of the diffusivity and viscous attenuation models is equally evident. While the linear solver tends to preserve excessive high-frequency content, leading to unrealistic spectral tails, the diffusivity and viscous models produce frequency responses that more closely replicate the experimental spectral roll-off, especially in the mid-to-high frequency range (2–5 MHz). The diffusivity model stands out for better reproducing the overall spectral slope across most tissue types, suggesting that it more effectively incorporates frequency-dependent loss mechanisms. In low-attenuation tissues such as adipose tissue and myocardium, both advanced models perform similarly well, whereas in structurally complex tissues like trabecular bone and cartilage, the diffusivity model maintains superior spectral fidelity. The instability observed in cortical bone, liver, and cartilage in the time domain is also reflected here, with minor spectral noise in the high-frequency tail.

In terms of computational cost, the linear solver remains the fastest method, requiring significantly less processing time than either of the nonlinear attenuation approaches. However, this speed advantage comes at the expense of accuracy, as evidenced in both the time and frequency domains. The viscous attenuation model generally requires the longest computation times due to the additional terms governing bulk and shear viscous effects, while the diffusivity model offers a balanced trade-off, achieving better accuracy than the linear solver while maintaining lower computation times than the viscous attenuation formulation. This balance may be particularly relevant for large-scale simulations where computation time is a limiting factor.

The coefficient of determination (R^2^) values further confirms the observations from the waveform and spectral plots. Across all tissue types, both the diffusivity and viscous attenuation models achieve high R^2^ values, often exceeding 0.99, indicating excellent agreement with the experimental data. The diffusivity model achieves the highest R^2^ in the majority of cases, reinforcing its advantage in replicating the experimental results. In contrast, the linear solver produces substantially lower R^2^ values, particularly in tissues with high attenuation, confirming its limitations in accurately modeling frequency-dependent losses. In tissues such as cortical bone, liver, and cartilage, where nonlinear solver instability was observed, the R^2^ values remained high but showed slightly more deviation compared to other tissues, suggesting that solver stability and parameter tuning will be important considerations for future studies.

#### 4.2.2. Acoustic Wave Propagation (5 Megahertz)

**Figure 10:**
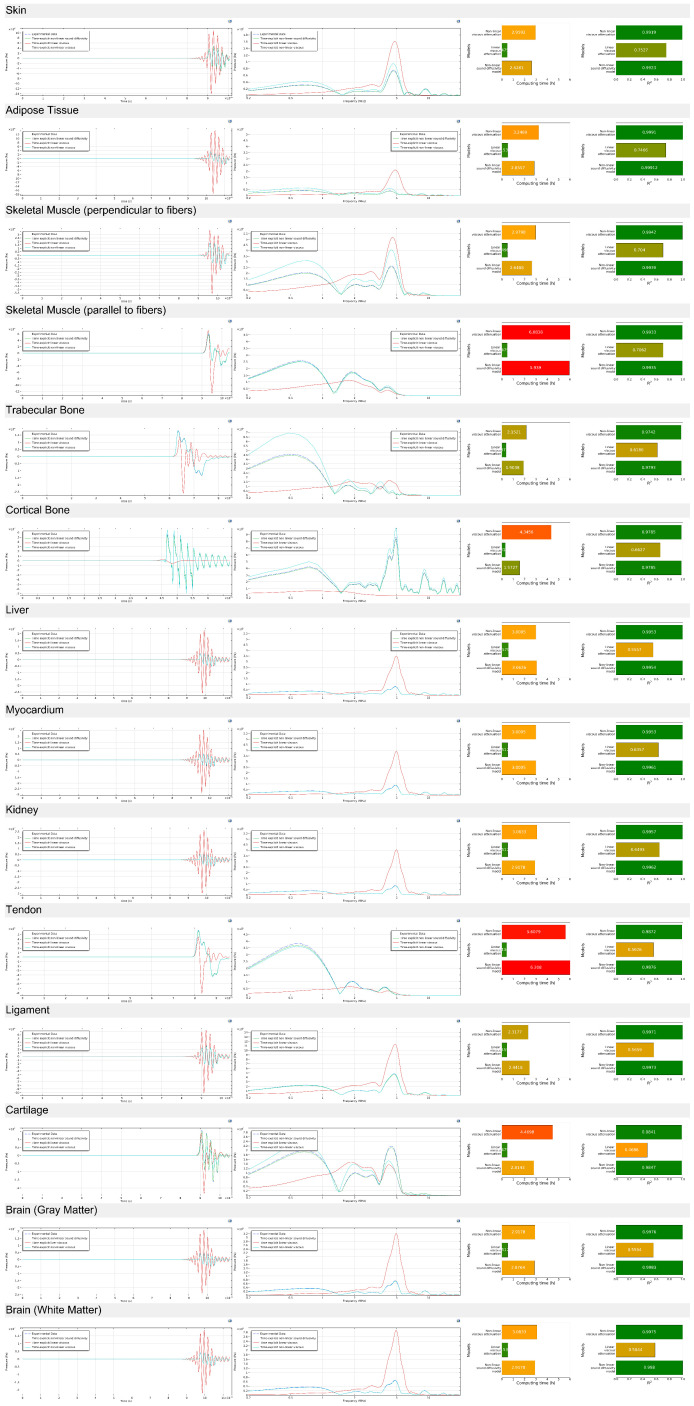
Time domain, frequency domain, computing time, and coefficient of determination (R^2^) of the studied samples at 5 MHz.

To further evaluate the attenuation and dispersion characteristics across different biological tissues, an additional set of simulations was performed for a 5 MHz acoustic wave, using the same metrics as in the previous analysis (Figure 10). This high-frequency case allows for assessing how dissipative mechanisms behave under shorter wavelengths and higher energy densities. For the sake of increasing readability and given that similar trends were consistently observed at other frequencies, the results for 1, 2, and 4 MHz have been moved to the Appendix A (Figure A16, Figure A17 and Figure A18). As before, a typically adopted linear solver was included for direct comparison with the proposed sound diffusivity and viscous attenuation models.

In the time-domain results, simulations at 5 MHz show a noticeably faster waveform decay compared to the lower-frequency case presented in the previous figure. This is consistent with the increased frequency-dependent attenuation observed across all tissues. The alignment between the simulated and experimental waveforms remains high for most tissues, although certain highly attenuating or structurally complex samples, particularly cortical bone, tendon, and skeletal muscle (parallel to fibers), exhibit small but visible discrepancies in the later cycles. These differences likely stem from stronger dispersive effects and higher attenuation rates at this frequency, which are more difficult to capture with perfect fidelity.

In the frequency domain, the amplitude spectra at 5 MHz exhibit broader attenuation profiles, with more significant suppression of high-frequency components compared to the lower-frequency results. This effect is especially pronounced in fluid-rich tissues such as liver and myocardium, where bulk viscosity-driven compressional damping dominates. Compared to the previous figure, these spectra highlight the more challenging nature of high-frequency propagation, where even small parameter estimation errors can lead to amplified deviations in the frequency domain.

Regarding computational time, both the sound diffusivity and nonlinear viscous models remain more computationally demanding than the baseline linear solver. However, at 5 MHz, the gap in computing time between the two advanced models widens compared to the lower-frequency simulations. The nonlinear viscous model, while maintaining very high accuracy, shows a proportionally larger increase in computational cost at this frequency. Conversely, the sound diffusivity approach continues to offer a good compromise, with strong accuracy and lower computational burden, making it an attractive option when high throughput or parameter sweeps are required.

The R^2^ metrics confirm that, even at 5 MHz, both advanced models significantly outperform the linear solver across all tissue types. However, the increased frequency seems to exacerbate modeling challenges for certain tissues—most notably cortical bone, tendon, and cartilage, where the nonlinear solver occasionally shows localized instability or underperformance. These differences were less pronounced in the previous figure at lower frequency. This suggests that, while both advanced models retain their superiority, the diffusivity-based approach offers a more stable and computationally efficient solution for high-frequency biomedical ultrasound modeling, especially in complex or high-attenuation tissues.

#### 4.2.3. Sound Diffusivity Regression

**Figure 11:**
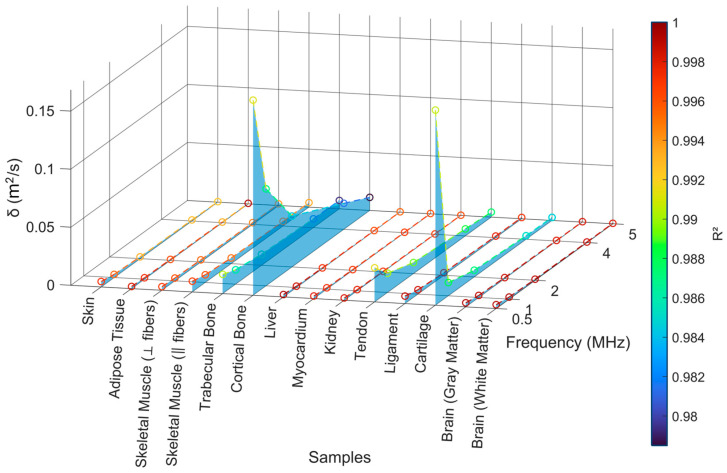
Frequency-dependent sound diffusivity (δ, m^2^/s) in biological tissues. Color scale indicates the R^2^ of the regression fit. The circles represent the obtained values.

Figure 11 presents the estimated sound diffusivity (*δ*) values across 13 biological tissues as a function of frequency (0.5–5 MHz), along with the corresponding coefficient of determination (R^2^) for the sound diffusivity parameter estimation. Table A5 specifies the calculated sound diffusivity for each frequency.

Overall, the results confirm that *δ* tends to decrease with frequency, as expected from theoretical models of frequency-dependent attenuation. Soft tissues such as skin (Figure A4A), adipose tissue (Figure A4B), brain (Figure A7A,B), and myocardium (Figure A5D) display consistently low *δ* values, typically below 0.005 m^2^/s, with excellent fitting accuracy (R^2^ > 0.995), validating the suitability of the power-law attenuation model for these media. The smooth trends and high R^2^ values suggest homogeneous acoustic behavior, dominated by viscoelastic damping rather than scattering.

Skeletal muscle (Figure A4C,D) also follows a similar trend, although its *δ* values vary slightly between fiber orientations. Both perpendicular and parallel fiber arrangements exhibit predictable frequency-dependent attenuation with high R^2^ (>0.99), indicating that despite its anisotropic structure, skeletal muscle conforms well to the power-law model. Similarly, liver (Figure A5C) demonstrates low *δ* values and near-perfect fitting accuracy (R^2^ ≈ 1), consistent with its homogeneous, fluid-rich microarchitecture.

In contrast, tendon (Figure A6B), kidney (Figure A6A), and ligament (Figure A6C) exhibit slightly higher *δ* values than the aforementioned soft tissues, particularly at lower frequencies. This likely reflects their fibrous structure (tendon, ligament) and complex vascularization and parenchymal heterogeneity (kidney), which contribute to more complex viscoelastic or scattering behaviors. Nonetheless, the regression quality remains high (R^2^ > 0.99), confirming that the power-law model remains a valid approximation.

In contrast, higher *δ* values are observed in trabecular (Figure A5A) and cortical bone (Figure A5B), especially at lower frequencies. This likely reflects complex interactions between compressional wave modes and microstructural scattering due to bone porosity and mineral content. Notably, the fit quality (R^2^) for these tissues decreases slightly in the higher-frequency range (4–5 MHz), indicating that the simulation framework struggles to accurately resolve the steep attenuation gradients observed in highly lossy media at elevated frequencies. This issue arises primarily due to numerical limitations: at standard CFL conditions (e.g., 0.2), which were sufficient for most other tissues, the solver occasionally exhibited unstable behavior, manifesting as an exponential surge in acoustic pressure, a clear sign of a numerical artifact likely caused by inadequate mesh or time resolution. To mitigate this, the CFL condition was reduced further, improving simulation fidelity at the cost of additional computation. Additionally, switching from the classic Runge–Kutta solver to the more stable RK34 (Runge–Kutta of order 3/4) significantly enhanced convergence. Although the Adams–Bashforth method was also tested for time integration, it led to increased computational time and a higher likelihood of instability, particularly in tissues with very high attenuation such as cortical bone, making it unsuitable for this application. These adjustments underscore the importance of solver selection and mesh/time-step calibration in simulating nonlinear acoustic wave propagation in high-attenuation media.

Interestingly, cartilage (Figure A6D) also exhibits elevated *δ* at low frequencies and slightly reduced R^2^, likely due to its biphasic structure and water content, which introduce nonlinear viscoelastic attenuation that deviates from idealized power-law behavior. Despite this, the regression remains acceptably high (R^2^ > 0.98), suggesting that while the power-law fit may not be exact, it still provides a robust approximation across most samples.

The plot also highlights that parameter estimation remained stable across all tissues and frequencies, with no values falling below R^2^ = 0.98. This reinforces the consistency of the simulation workflow and the validity of the inverse modeling approach to extract sound diffusivity from experimental attenuation data.

**Figure 12:**
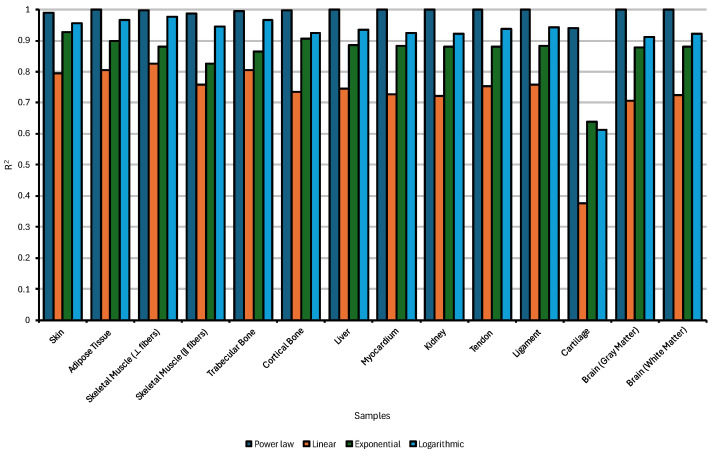
Goodness-of-fit (R^2^) of four regression models (power law, linear, exponential, logarithmic) applied to sound diffusivity data across biological tissues.

The comparative analysis of regression models applied to the estimation of sound diffusivity is shown in Figure 12 and explored in more detail in Appendix B.1.

Across various biological tissues it is revealed that the power-law model consistently outperforms linear, exponential, and logarithmic fits, as evidenced by its higher coefficients of determination (R^2^) across all samples. This suggests that the underlying relationship between sound diffusivity and the relevant independent variables in biological tissues is inherently nonlinear, favoring scale-invariant dynamics characteristic of power-law behavior. Notably, tissues such as cortical bone, ligament, cartilage, and liver exhibited R^2^ values exceeding 0.98 under the power-law model, highlighting its suitability for modeling the complex propagation of ultrasound in structurally anisotropic or poroelastic media. In contrast, the linear regression model systematically underperformed, with R^2^ values frequently falling below 0.85, particularly in soft tissues like brain gray matter and skeletal muscle, where heterogeneity and high water content introduce significant nonlinear interactions. While exponential and logarithmic regressions provided intermediate fits, they generally lacked the accuracy and consistency of the power law, especially in capturing the intricacies of wave-tissue interactions in neural and musculoskeletal structures. Cartilage exhibited comparatively lower R^2^ values across all regression models, indicating that existing approaches may be insufficient to fully capture the complexity of ultrasound diffusion in this tissue; thus, alternative modeling techniques or experimental methodologies may be required to more accurately quantify its acoustic behavior. These findings reinforce the importance of adopting nonlinear modeling frameworks in biomedical acoustics, where tissue-specific viscoelastic, porous, and anisotropic properties critically influence ultrasonic energy diffusion.

As such, the *δ* of biological tissues can be explained by a power-law formulation, according to Equation (19),

(19) $$\delta (f)=\delta_{0}f^{n}$$

where δ(f) is the sound diffusivity ($m^{2}/s$) at frequency f (MHz), $\delta_{0}$ is the reference diffusivity constant at *f* = 1 MHz, and n is the frequency exponent, a material-dependent parameter that shows how strongly the diffusivity increases or decreases with frequency. The suggested parameters for each sample can be found in Table 5.

To the best of the authors’ knowledge, no prior studies have reported experimental values of sound diffusivity for the biological tissues examined in this work. While attenuation and propagation speed have been extensively documented in literature, the frequency-dependent behavior of sound diffusivity, particularly derived from experimental data and validated through numerical modeling, remains absent from current scientific discourse. As such, the findings presented here offer a novel contribution, expanding the acoustic characterization of biological media and providing critical parameters for future computational and therapeutic applications.

In summary, the study of sound diffusivity across biological tissues underscores the fundamental importance of frequency-dependent acoustic dissipation in biomechanical modeling. The consistent superiority of the power-law model (Equation (16)), in fitting experimental data across a wide range of tissues demonstrates its reliability and physical relevance. These experimentally derived parameters serve as essential inputs for numerical simulations, ensuring that computational models reflect the true nature of wave propagation within complex biological media. This study thus bridges experimental insight with simulation accuracy, laying the groundwork for advanced predictive modeling in both therapeutic and diagnostic ultrasound. In essence, understanding sound diffusivity is not simply a technical necessity, it is a vital step toward simulating the physiological reality of tissue behavior under acoustic excitation.

#### 4.2.4. Viscous Attenuation Regression

**Figure 13:**
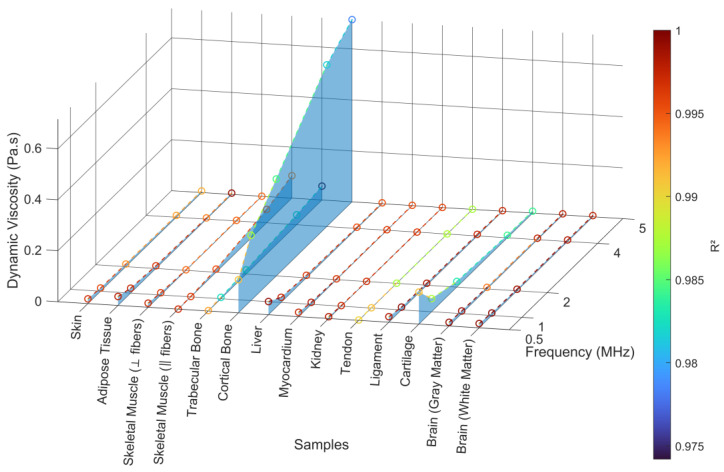
Frequency-dependent dynamic viscosity (Pa·s) of biological tissues obtained from viscous attenuation simulations. Color scale indicates the R^2^ of the model fit. The circles represent the obtained values at each frequency.

Figure 13 presents the estimated dynamic viscosity values across 13 biological tissues as a function of frequency (0.5–5 MHz), along with the corresponding coefficient of determination (R^2^). Table A6 also specifies the calculated dynamic viscosity for each frequency.

Dynamic viscosity, a fundamental viscoelastic parameter, quantifies the internal friction arising from transverse (shear) deformation, and its role becomes particularly relevant in tissues where shear wave propagation or complex internal structure may modulate acoustic behavior. Across the investigated frequency range (0.5–5 MHz), most tissues exhibited low dynamic viscosity values, typically below 0.05 Pa·s. Slightly elevated values were observed in structurally organized tissues such as skeletal muscle (‖ fibers; Figure A8D) and ligament (Figure A10C), which increase their resistance to transverse motion. Tendon (Figure A10B) also displayed comparable shear damping characteristics, though to a slightly lesser extent. These patterns are consistent with the known biomechanical behavior of fibrous connective tissues, which often display frequency-dependent viscoelasticity under shear deformation.

In contrast, skin (Figure A8A), adipose tissue (Figure A8B), skeletal muscle (⟂ fibers; Figure A8C), and trabecular bone (Figure A9A) showed modest but clearly defined frequency-dependent viscosity, suggesting intermediate viscoelastic damping behavior, likely due to their composite architecture and partially ordered structures [51]. Cortical bone (Figure A9B), being highly mineralized and rigid, exhibited negligible dynamic viscosity across all frequencies, as expected from a medium where shear-related dissipation is largely suppressed.

Liver (Figure A9C) and myocardium (Figure A9D), while not exhibiting high dynamic viscosity values, demonstrated exceptionally consistent regression fits (R^2^ ≈ 1), indicating a predictable and well-modeled attenuation trend. This behavior may be attributed to their homogeneous viscoelastic structure and highly vascularized parenchyma, which facilitate stable shear response across frequencies [96,97]. Similarly, kidney (Figure A10A) demonstrated a smooth, low-viscosity profile with high R^2^ values, consistent with its soft, isotropic tissue composition [98].

Conversely, cartilage (Figure A10D), brain gray matter (Figure A11A), and brain white matter (Figure A11B) displayed very low dynamic viscosity values accompanied by slightly reduced R^2^ values, suggesting that simple shear viscosity models may not adequately capture their acoustic attenuation behavior. In these tissues, attenuation may instead be dominated by poroelastic diffusion, scattering from microscopic inhomogeneities, or frequency-dependent anisotropy, especially in the highly hydrated and structurally complex environments of brain and cartilage [99,100]. These findings underscore the tissue-specific relevance of dynamic viscosity and highlight the need for advanced multiphysics modeling approaches in acoustically heterogeneous or anisotropic biological media.

**Figure 14:**
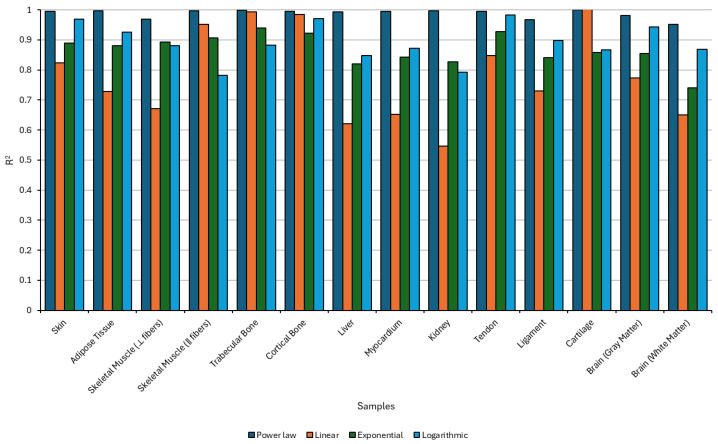
Goodness-of-fit (R^2^) for four regression models applied to dynamic viscosity data across biological tissues.

The regression analysis presented in the bar chart evaluates the predictive accuracy of four functional models (power law, linear, exponential, and logarithmic) in fitting the dynamic viscosity of various biological tissues (Figure 14).

The power-law model consistently outperformed the other regression types, achieving the highest R^2^ values across nearly all tissue types, indicating its strong suitability for describing the frequency-dependent behavior of dynamic viscosity in soft and hard tissues. This result aligns with the expected nonlinear rheological response of biological media, where shear-thinning and frequency-dependent dissipation often follows scale-invariant patterns. Tissues such as skin, cortical bone, tendon, and liver showed particularly robust fit with the power law, reinforcing its appropriateness in modeling viscoelastic or poroelastic attenuation mechanisms.

While the logarithmic and exponential models occasionally approached similar levels of fit (especially in tissues like ligament and myocardium) they exhibited greater variability and generally lower predictive accuracy. The linear model, on the other hand, demonstrated the least reliability, with markedly lower R^2^ values in tissues such as skeletal muscle, gray matter, and cartilage, confirming that a simple linear relationship does not adequately describe the complex viscoelastic properties of these biological materials.

Of particular note are the relatively low R^2^ values observed for cartilage and brain tissues, regardless of the model used. This suggests that the relationship between frequency and viscosity in these samples is either governed by non-conventional mechanisms (e.g., osmo/visco/poroelasticity, microstructural heterogeneity, or fluid–solid coupling) or requires more advanced modeling frameworks beyond standard regression forms. These results highlight the importance of selecting tissue-appropriate mathematical models and may motivate the development of hybrid or multiparametric approaches to more accurately capture acoustic attenuation in highly heterogeneous or structurally complex biological environments.

The dynamic viscosity (μ) was modeled as a function of ultrasonic frequency (*f*) according to Equation (20),

(20) $$\mu (f)=\mu_{0}f^{n}$$

where μ(f) represents the dynamic viscosity at a given frequency *f*, $\mu_{0}$ is the reference viscosity at 1 MHz, and *n* is the frequency exponent, which captures how viscosity evolves across the frequency spectrum. A negative value of *n* indicates that viscosity decreases with increasing frequency, a behavior commonly observed in biological soft tissues due to shear-thinning or viscoelastic relaxation phenomena, where internal structural resistance diminishes at higher deformation rates [101]. The estimated parameters are presented in Table 6.

**Figure 15:**
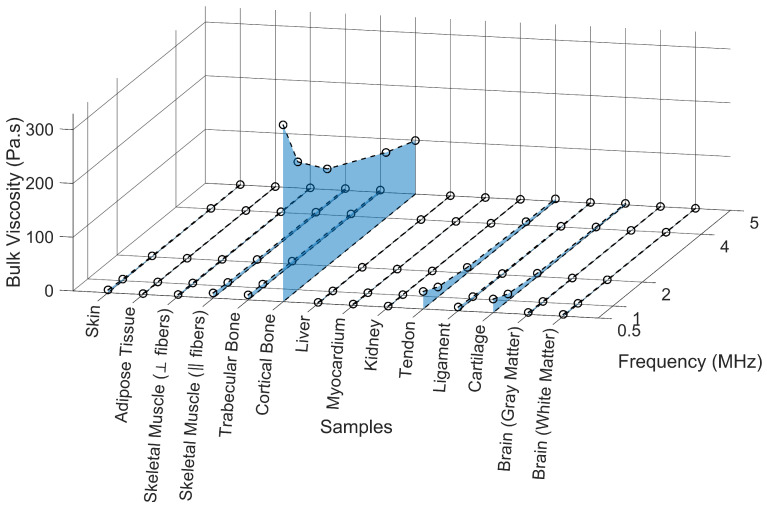
Frequency-dependent bulk viscosity (Pa·s) in biological tissues.

Figure 15 presents the estimated bulk viscosity across the studied frequencies by sample, along with the corresponding coefficient of determination (R^2^) for the sound diffusivity parameter estimation. Table A7 specifies the calculated bulk viscosity for each frequency.

Bulk viscosity quantifies the resistance of a medium to volumetric deformation, and unlike dynamic (shear) viscosity, it directly influences the attenuation of longitudinal acoustic waves, which are the dominant mode in most ultrasound applications. Among all samples, cortical bone (Figure A13B) clearly exhibits the highest bulk viscosity values, reaching over 300 Pa·s at lower frequencies, which is markedly greater than any soft tissue observed. This elevated bulk viscosity in cortical bone suggests a significant resistance to volumetric strain, potentially due to its dense mineralized matrix and low porosity, which hinder internal fluid motion and amplify energy loss during compressional loading. These results emphasize that even in acoustic stiff media, such as bone, bulk damping mechanisms can be prominent, particularly under the high-frequency stress conditions modeled here.

Several other tissues like skeletal muscle (Figure A13C,D), trabecular bone (Figure A13A), tendon (Figure A14B), ligament (Figure A14C), and cartilage (Figure A14D) also exhibited modest bulk viscosity values, especially at low frequencies, before declining with increasing frequency. This behavior aligns with their partially organized microstructures and moderate resistance to compressive deformation [76,102,103], which can result in observable but less dominant volumetric losses. In these tissues, internal fluid redistribution, fiber alignment, and matrix porosity are likely to contribute to measurable bulk damping, although to a far lesser extent than in cortical bone.

Notably, liver (Figure A13C) and myocardium (Figure A13D) exhibit exceptionally elevated bulk viscosity values, exceeding 200 Pa·s at low frequencies (0.5–1 MHz), with a gradual decline observed as frequency increases. This trend suggests that these highly vascularized and metabolically active organs undergo substantial compressional relaxation, likely due to their rich interstitial fluid content, dense extracellular matrix, and viscoelastic cellular structure. Such findings are consistent with theoretical expectations from poroviscoelastic frameworks [97,104] and corroborate prior reports highlighting the importance of compressive damping in fluid-rich tissues.

However, despite the high values observed in these organs, other parenchymal tissues such as skin (Figure A12A), adipose tissue (Figure A12B), and kidney (Figure A14A), maintained consistently low bulk viscosity values across all frequencies. In skin, this could be due to its laminar structure and barrier function, while in adipose tissue, the low compressive resistance of lipid-filled adipocytes likely limits bulk damping potential.

Finally, brain gray (Figure A15A) and white matter (Figure A15B) demonstrated uniformly low bulk viscosity, consistent with their high water content, softness, and anisotropic mechanical behavior [105], which do not significantly resist compressional deformation. These results suggest that compressional damping plays a minimal role in attenuation for most soft, compliant tissues, where shear viscosity, scattering, or poroelastic coupling are likely more influential.

Altogether, this analysis reveals the tissue-specific nature of bulk viscosity, underlining its importance in simulations involving high-impedance transitions (e.g., bone-soft tissue interfaces) or in predicting localized heating and stress distributions in therapeutic ultrasound. Accurate incorporation of this parameter is essential to improving the realism of computational models, particularly in scenarios involving heterogeneous anatomical structures or nonlinear propagation regimes.

**Figure 16:**
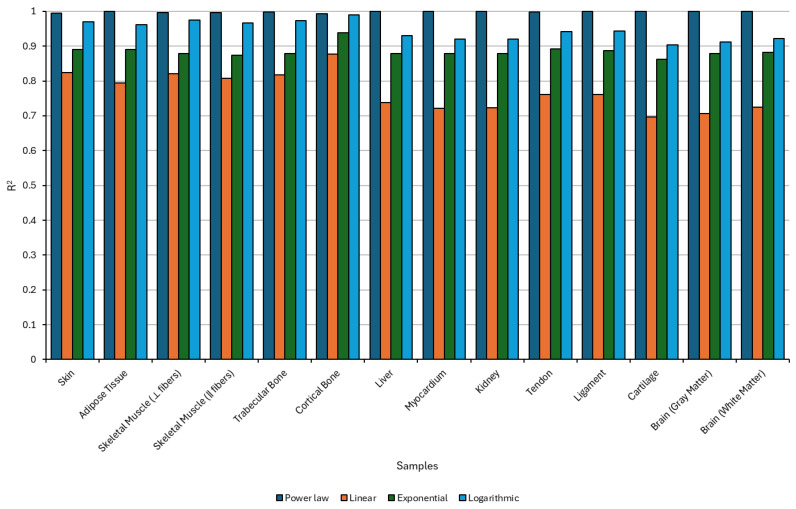
Goodness-of-fit (R^2^) of four regression models applied to bulk viscosity data across biological tissues.

**Figure A1:**
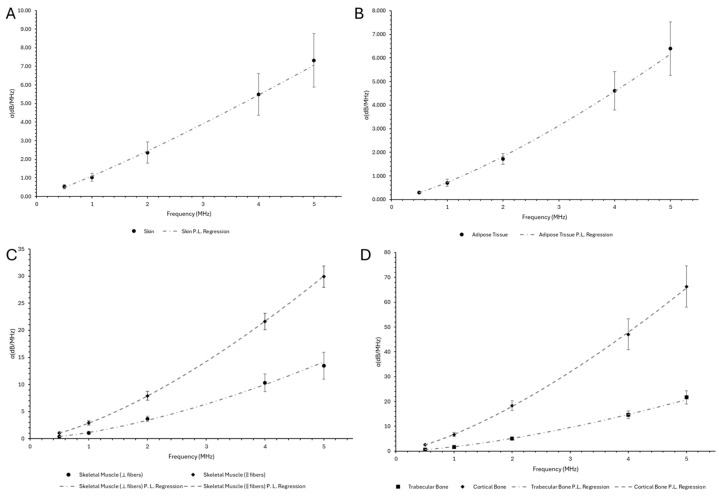
Frequency-dependent attenuation coefficient (*α*) for (**A**) skin, (**B**) adipose tissue, (**C**) skeletal muscle, and (**D**) bone with power-law regression fit. Error bars represent standard deviation across measurements.

**Figure A2:**
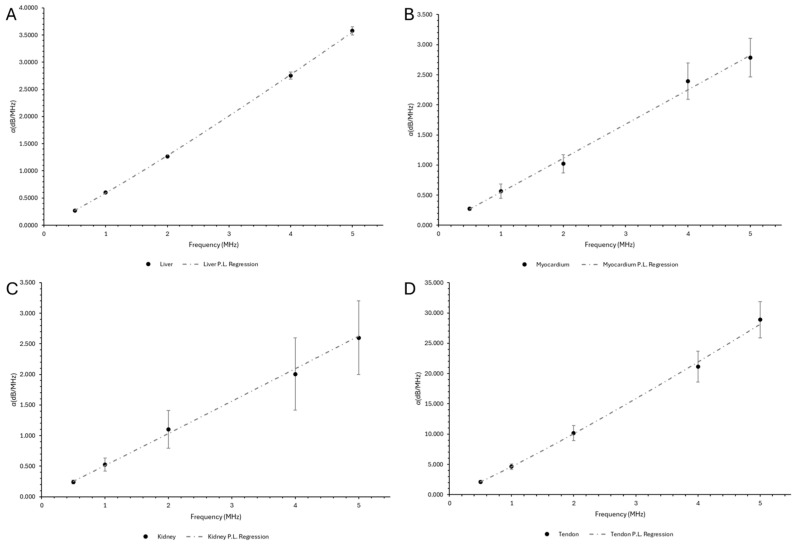
Frequency-dependent attenuation coefficient (*α*) for (**A**) liver, (**B**) myocardium, (**C**) kidney, and (**D**) tendon, with power-law regression fit. Error bars represent standard deviation across measurements.

**Figure A3:**
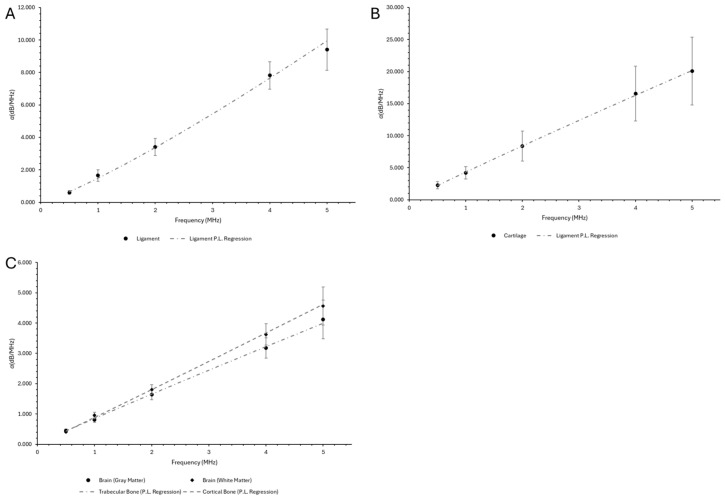
Frequency-dependent attenuation coefficient (*α*) for (**A**) ligament, (**B**) cartilage, and (**C**) brain (gray and white matter), with power-law regression fit. Error bars represent standard deviation across measurements.

**Figure A4:**
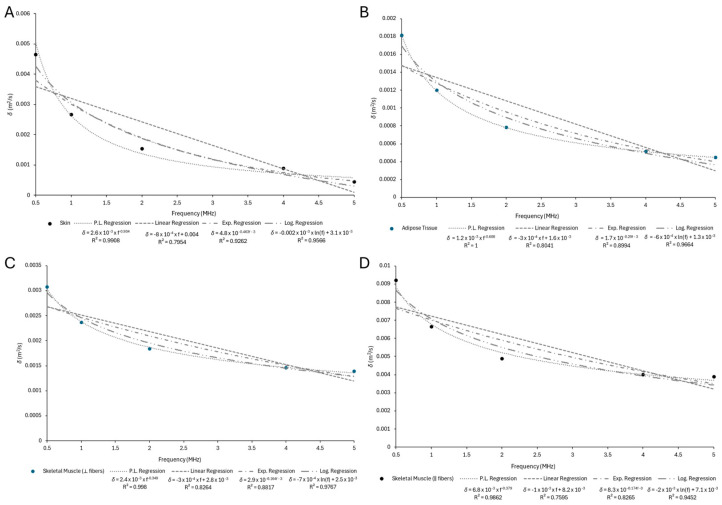
Frequency-dependent behavior of the sound diffusivity (*δ*) in (**A**) skin, (**B**) adipose tissue, (**C**) skeletal muscle (⊥ fibers), and (**D**) skeletal muscle (‖ fibers), analyzed using four different regression models: power law, linear, exponential, and logarithmic.

**Figure A5:**
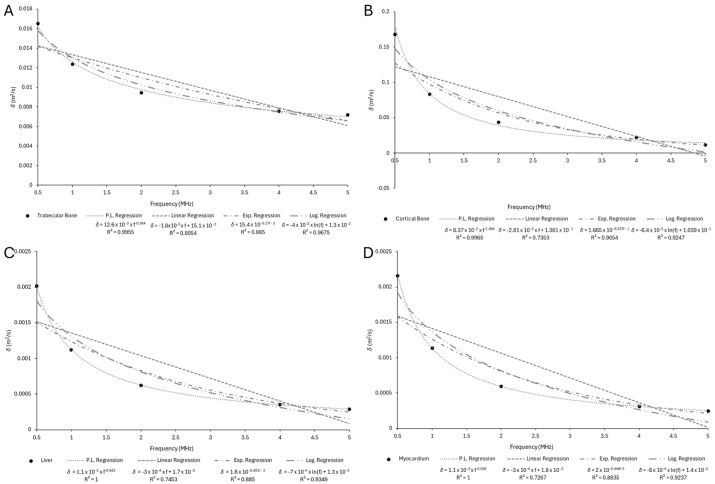
Frequency-dependent behavior of the sound diffusivity (*δ*) in (**A**) trabecular bone, (**B**) cortical bone, (**C**) liver, and (**D**) myocardium, analyzed using four different regression models: power law, linear, exponential, and logarithmic.

**Figure A6:**
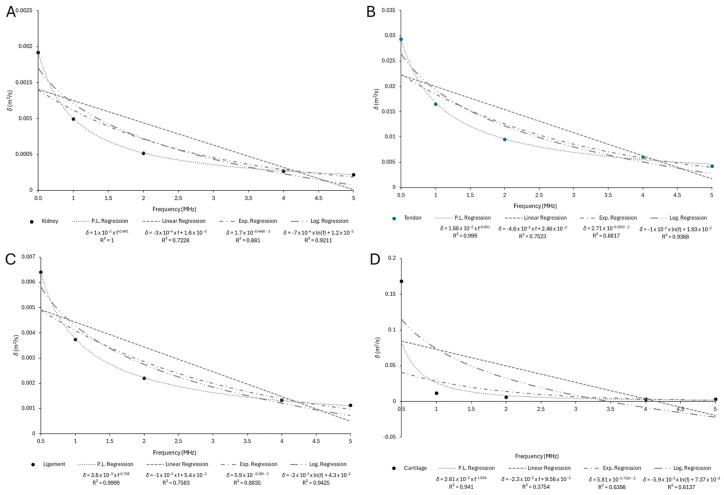
Frequency-dependent behavior of the sound diffusivity (*δ*) in (**A**) kidney, (**B**) tendon, (**C**) ligament, and (**D**) cartilage, analyzed using four different regression models: power law, linear, exponential, and logarithmic.

**Figure A7:**
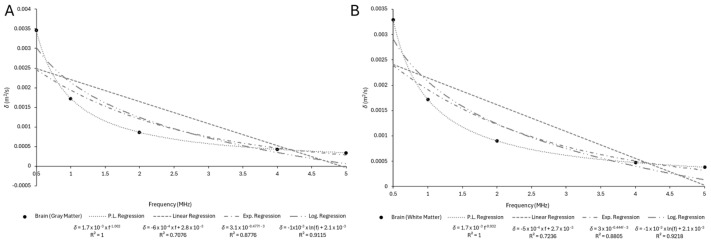
Frequency-dependent behavior of the sound diffusivity (*δ*) in (**A**) brain (gray matter) and (**B**) brain (white matter), analyzed using four different regression models: power law, linear, exponential, and logarithmic.

**Figure A8:**
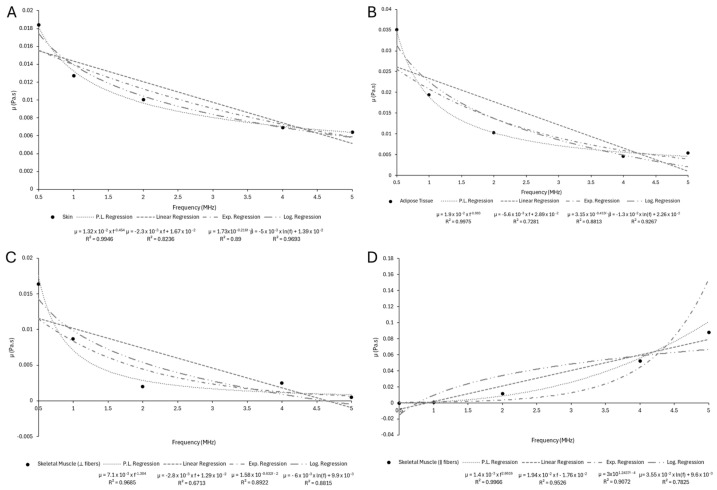
Frequency-dependent behavior of dynamic viscosity in (**A**) skin, (**B**) adipose tissue, (**C**) skeletal muscle (⊥ fibers), and (**D**) skeletal muscle (‖ fibers), analyzed using four different regression models: power law, linear, exponential, and logarithmic.

**Figure A9:**
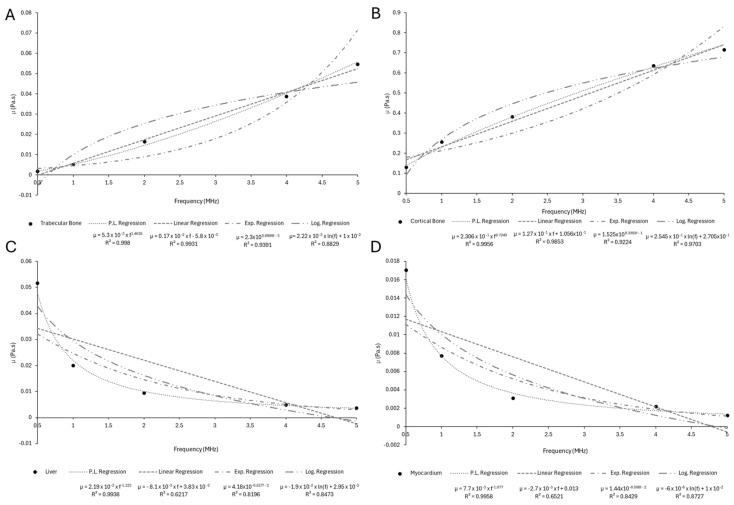
Frequency-dependent behavior of dynamic viscosity in (**A**) trabecular bone, (**B**) cortical bone, (**C**) liver, and (**D**) myocardium, analyzed using four different regression models: power law, linear, exponential, and logarithmic.

**Figure A10:**
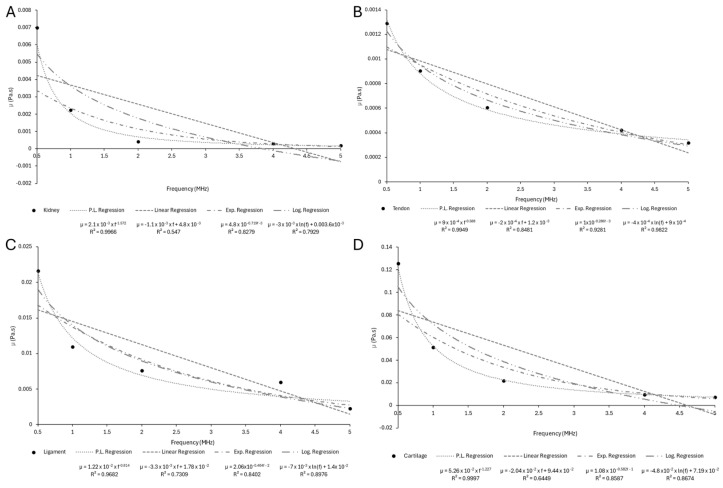
Frequency-dependent behavior of dynamic viscosity in (**A**) kidney, (**B**) tendon, (**C**) ligament, and (**D**) cartilage, analyzed using four different regression models: power law, linear, exponential, and logarithmic.

**Figure A11:**
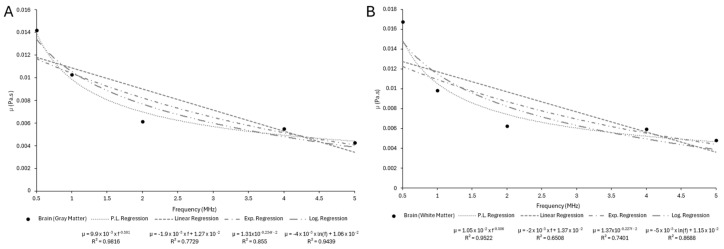
Frequency-dependent behavior of dynamic viscosity in (**A**) brain (gray matter) and (**B**) brain (white matter), analyzed using four different regression models: power law, linear, exponential, and logarithmic.

**Figure A12:**
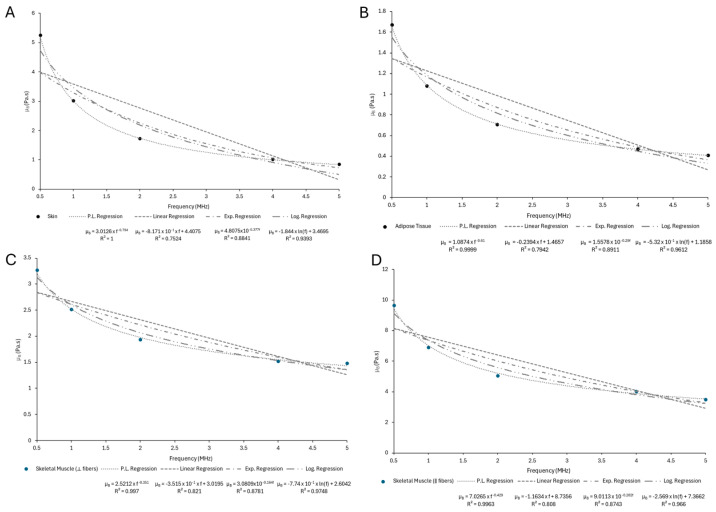
Frequency-dependent behavior of bulk viscosity in (**A**) skin, (**B**) adipose tissue, (**C**) skeletal muscle (⊥ fibers), and (**D**) skeletal muscle (‖ fibers), analyzed using four different regression models: power law, linear, exponential, and logarithmic.

**Figure A13:**
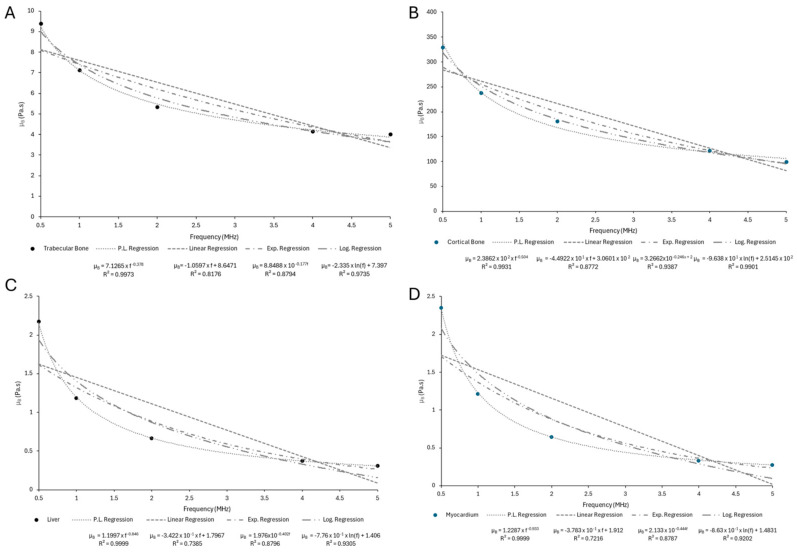
Frequency-dependent behavior of bulk viscosity in (**A**) trabecular bone, (**B**) cortical bone, (**C**) liver, and (**D**) myocardium, analyzed using four different regression models: power law, linear, exponential, and logarithmic.

**Figure A14:**
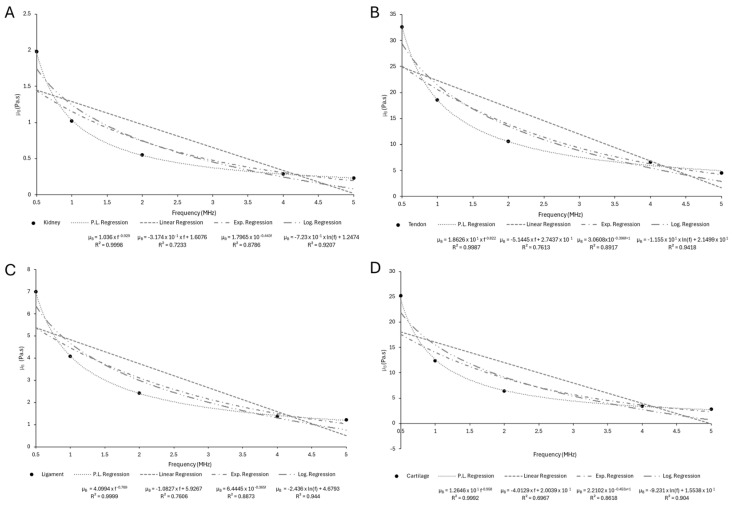
Frequency-dependent behavior of bulk viscosity in (**A**) kidney, (**B**) tendon, (**C**) ligament, and (**D**) cartilage, analyzed using four different regression models: power law, linear, exponential, and logarithmic.

**Figure A15:**
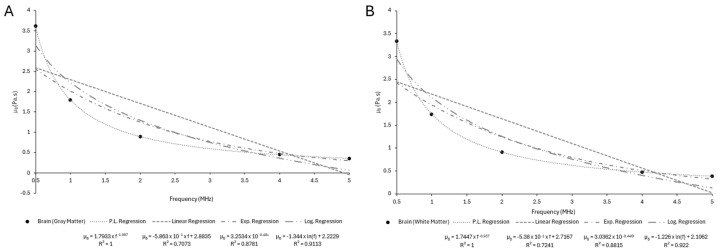
Frequency-dependent behavior of bulk viscosity in (**A**) brain (gray matter) and (**B**) brain (white matter), analyzed using four different regression models: power law, linear, exponential, and logarithmic.

**Figure A16:**
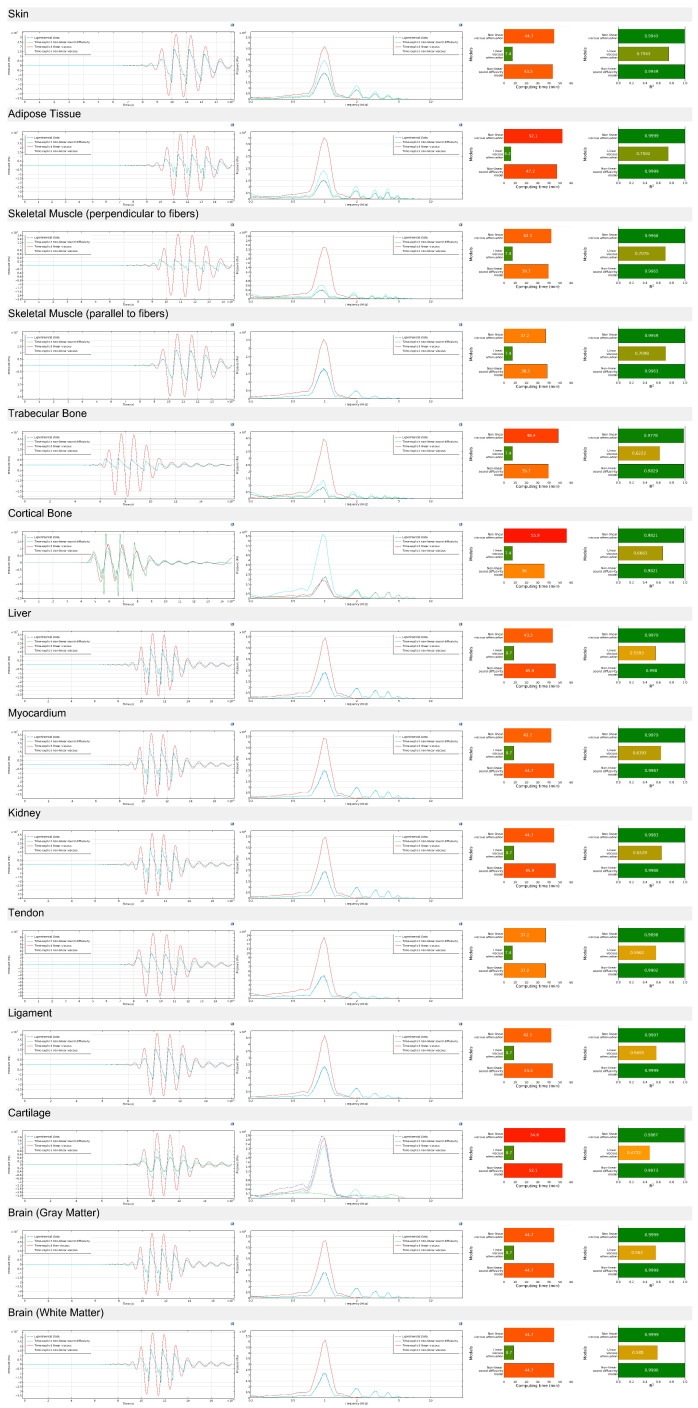
Time domain, frequency domain, computing time, and coefficient of determination (R^2^) of the studied samples at 1 MHz.

**Figure A17:**
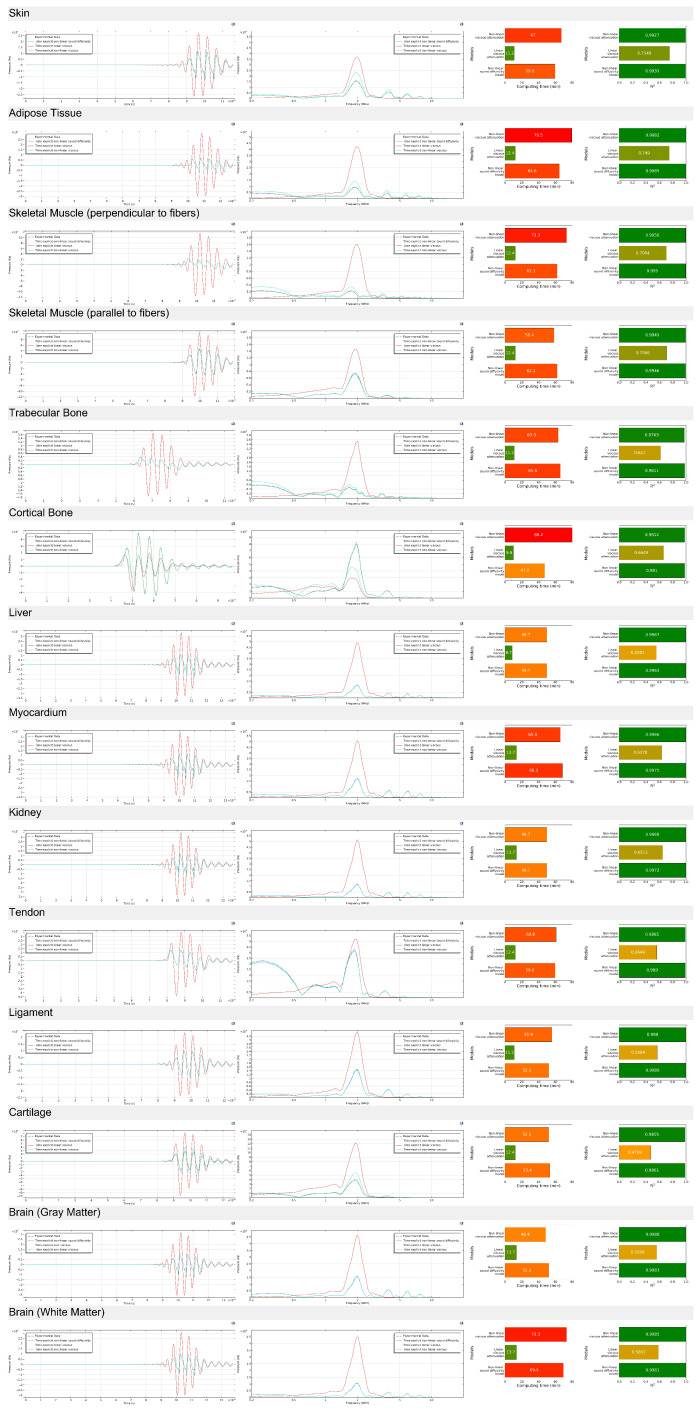
Time domain, frequency domain, computing time, and coefficient of determination (R^2^) of the studied samples at 2 MHz.

**Figure A18:**
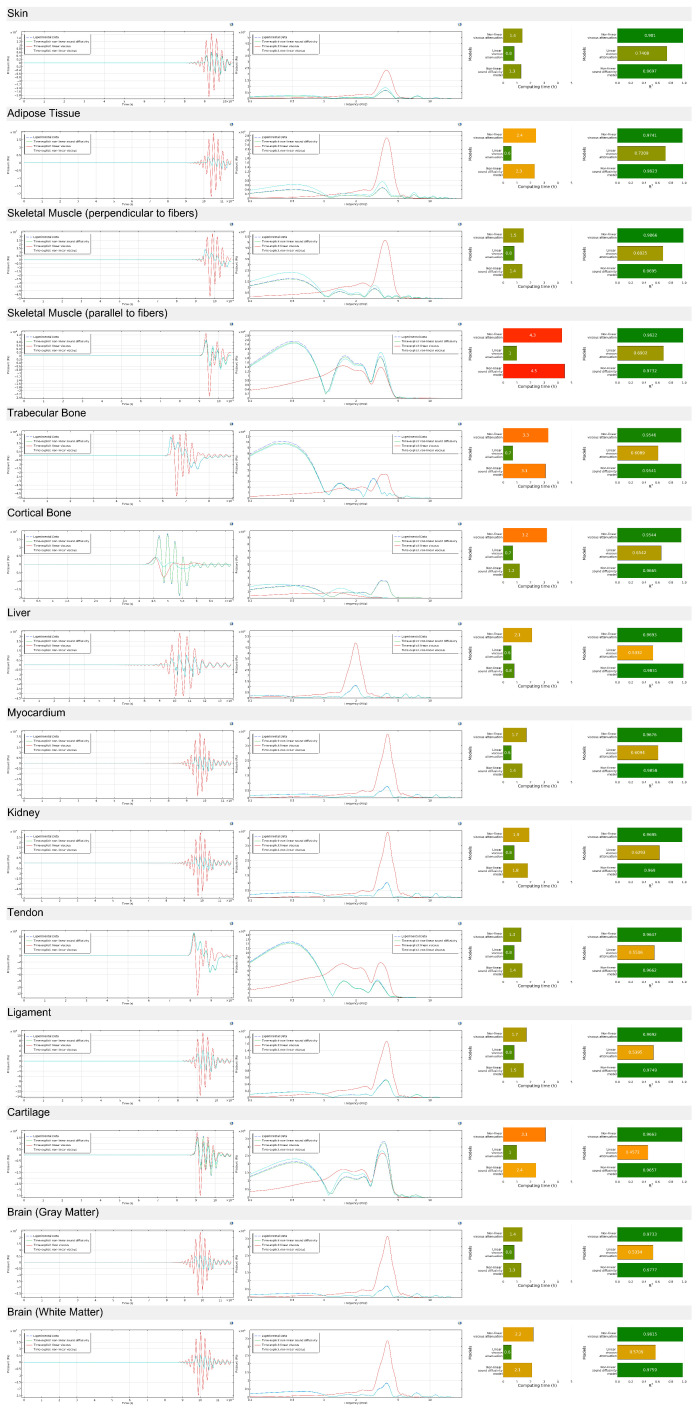
Time domain, frequency domain, computing time, and coefficient of determination (R^2^) of the studied samples at 4 MHz.

To evaluate the frequency-dependent behavior of bulk viscosity in biological tissues, four previously used regression models were applied to the simulated data. The coefficient of determination (R^2^) was used to assess the fit quality for each tissue and regression type, and is displayed in Figure 16.

Among the tested models, the power law consistently achieved the highest R^2^ values across nearly all tissue types, often approaching 1, thus indicating a superior ability to capture the nonlinear relationship between ultrasound frequency and bulk viscosity. This result strongly supports the hypothesis that compressive viscous dissipation in biological tissues follows a scale-invariant, frequency-dependent behavior, aligning with theoretical expectations from continuum mechanics and soft tissue rheology.

Tissues such as skin, cortical bone, tendon, ligament, and myocardium exhibited particularly robust fits under the power-law model, suggesting that their bulk viscous response to ultrasound propagation is well described by a frequency-scaling framework. The logarithmic model also demonstrated good predictive capacity, although generally trailing the power law, while exponential and linear models yielded lower R^2^ values in most cases, particularly for cartilage, brain tissues, and skeletal muscle, where complex relaxation mechanisms likely deviate from simple exponential decay or linear scaling assumptions.

The relatively poor performance of the linear model reinforces the inadequacy of oversimplified approaches for modeling bulk viscosity in acoustically heterogeneous or biologically intricate media. Notably, the regression fit for brain gray matter and cartilage (while still relatively high) revealed slightly lower R^2^ values even under the best-fitting models, hinting at underlying phenomena not captured by standard regression forms. These may include multiscale poroelasticity, fluid–solid coupling, or nonlinear viscoelastic relaxation, which would necessitate more advanced constitutive modeling to fully characterize.

The regression analysis validates the power-law model as the most reliable mathematical descriptor of frequency-dependent bulk viscosity in biological tissues. The findings underscore the need to adopt nonlinear and tissue-specific approaches when modeling compressive damping mechanisms in biomedical acoustics, particularly in the context of diagnostic imaging, therapeutic ultrasound, and tissue characterization frameworks.

The bulk viscosity, denoted as $\mu_{B}$, was expressed as a function of frequency (*f*) using Equation (21),

(21) $$\mu_{B}(f)=\mu_{B,0}f^{n}$$

where $\mu_{B}(f)$ represents the bulk viscosity at a given frequency f, $\mu_{B,0}$ is the reference bulk viscosity at 1 MHz, and *n* is the frequency exponent that characterizes how the bulk viscous damping scales across the ultrasonic spectrum. A positive value of *n* indicates increasing compressional losses with frequency, while a negative exponent would suggest diminishing contributions at higher frequencies. Table 7 exposes the suggested parameters.

In the conducted simulations, the attenuation of ultrasonic waves was observed to be more sensitive to variations in bulk viscosity than to dynamic (shear) viscosity. This heightened sensitivity arises from the nature of ultrasonic wave propagation in biological media, where compressional (longitudinal) waves dominate, and thus energy dissipation due to volumetric deformation plays a more pronounced role than shear-related mechanisms. While dynamic viscosity governs the attenuation of transverse particle motion and is relevant in shear wave modes or boundary-layer phenomena, bulk viscosity directly influences the rate of energy loss during compressive expansion and contraction cycles, which are intrinsic to acoustic pressure waves in soft tissues. This effect is amplified in tissues with high water content, dense cellular matrices, and low structural rigidity, where rapid fluid redistribution and compressive relaxation contribute significantly to wave damping. As such, the simulation results underscore the dominant role of bulk viscosity in modeling frequency-dependent attenuation in biologically relevant conditions and highlight the need to incorporate accurate compressive damping parameters when predicting acoustic energy deposition in therapeutic and diagnostic applications.

In future studies, greater emphasis should be placed on investigating shear wave propagation to more accurately quantify dynamic viscosity in biological tissues. While bulk viscosity predominantly governs the attenuation of compressional waves, dynamic viscosity is intrinsically linked to shear deformation and transverse wave motion, which are not fully captured in simulations focused solely on longitudinal acoustic modes. To isolate and characterize shear-related dissipative effects, experimental and computational frameworks must incorporate shear wave generation, propagation, and detection, ideally using modalities such as acoustic radiation force impulse (ARFI) imaging, shear wave elastography, or magneto-acoustic coupling. These techniques can provide spatially resolved data on tissue stiffness and viscoelastic damping, enabling more precise estimation of dynamic viscosity parameters. Furthermore, simulating shear wave behavior using viscoelastic or poroelastic models (validated against experimental benchmarks) would enhance our understanding of anisotropic and frequency-dependent shear attenuation, particularly in structured tissues such as cartilage, muscle, and ligament. Incorporating this dimension into future work will offer a more comprehensive viscoacoustic profile of tissues and strengthen the predictive power of ultrasound-based diagnostic and therapeutic modeling.

### 4.3. General Discusion

Both implemented models demonstrated consistently high accuracy (R^2^ > 0.99) when compared with the typically used linear solver, achieving closer agreement with experimental waveforms in both the time and frequency domains. While these advanced models are more computationally demanding due to the inclusion of frequency-dependent loss mechanisms and nonlinear terms, the gain in predictive capability justifies the added cost. Among them, the sound diffusivity model offered the best balance between accuracy and computational efficiency, providing markedly improved results over the linear solver while avoiding the higher computational expense associated with the viscous attenuation model. Their superior performance across a wide range of tissue types highlights their potential as more reliable alternatives to standard approaches, particularly in applications where model fidelity is critical for treatment planning, device optimization, or the accurate interpretation of ultrasound measurements.

The analysis of acoustic attenuation through both sound diffusivity and viscous modeling (via dynamic and bulk viscosity) estimation reveals complementary insights into the dissipative behavior of biological tissues under ultrasonic excitation. While sound diffusivity quantifies the overall rate of energy dispersion from an acoustic wavefront (accounting for both scattering and absorption phenomena), it provides a macroscopic, frequency-dependent metric that is particularly effective for capturing attenuation in heterogeneous or structurally complex tissues such as cartilage and brain. In contrast, the viscous attenuation models offer a more mechanistic breakdown of energy dissipation: dynamic viscosity governs shear-induced losses arising from transverse particle motion, whereas bulk viscosity captures compressive losses due to volumetric strain. The simulation results indicate that attenuation is more sensitive to bulk viscosity, especially in fluid-rich tissues like liver and myocardium, highlighting the dominance of compressive damping in the ultrasonic regime. However, the estimation of dynamic viscosity was less consistent across tissues, suggesting that shear wave propagation must be better resolved to refine this parameter. Meanwhile, the sound diffusivity regressions, particularly the power-law model, demonstrated strong predictive power across a broad tissue spectrum, making it a reliable tool for global attenuation characterization. Ultimately, combining diffusivity-based regression with tissue-specific viscous modeling provides a more comprehensive framework for understanding and predicting ultrasound-tissue interactions, and future studies should aim to integrate both approaches across a wider frequency spectrum to enhance the fidelity of biomechanical and therapeutic modeling.

Compared to all other tissues studied, cortical bone exhibited distinct acoustic and viscoacoustic properties which can be attributed to its highly mineralized composition, dense microstructure, and very low porosity [60,85,90]. These characteristics result in a substantially higher acoustic impedance and speed of sound, as well as elevated bulk viscosity, reflecting its strong resistance to both shear and compressional deformation. Unlike soft tissues, where attenuation is often dominated by viscous and scattering mechanisms mediated by interstitial fluids [70,106], energy dissipation in cortical bone arises primarily from scattering at microscopic mineral interfaces and intrinsic absorption within the rigid matrix. Furthermore, its organized lamellar structure and low water content reduce fluid-mediated relaxation processes, further distinguishing its acoustic behavior from that of hydrated, compliant tissues. These structural and compositional differences explain why cortical bone consistently shows values that are well outside the range observed for other biological tissues.

To further validate the estimated parameters and the robustness of their corresponding power-law regressions, future studies should extend the analysis to include both higher and intermediate frequency ranges beyond those currently explored. While the present frequency window (0.5–5 MHz) captures clinically relevant diagnostic and therapeutic domains, biological tissues exhibit frequency-dependent dispersion and attenuation behavior that may evolve significantly outside this band. Investigating higher frequencies (e.g., >10 MHz) would provide deeper insight into microstructural interactions, such as scattering and relaxation phenomena occurring at subcellular scales, which may reveal nonlinearities not fully captured at lower frequencies. Similarly, a more detailed examination of intermediate frequencies (1–3 MHz), where transitions between different attenuation mechanisms often occur, could help refine the fitting parameters and detect subtle deviations from idealized power-law scaling. By expanding the spectral range, researchers can improve the statistical robustness and physiological relevance of the fitted models, ensure better extrapolation accuracy, and ultimately enhance the predictive capability of simulation frameworks for diverse biomedical applications.

This study provides a multifaceted characterization of acoustic attenuation in biological tissues by integrating diffusivity analysis with viscous dissipation modeling. The findings underscore the necessity of adopting tissue-specific and mechanism-informed approaches when modeling ultrasound propagation, particularly for therapeutic and diagnostic applications. While power-law regressions proved robust across diffusivity and viscosity estimations, their underlying physical meaning varies with tissue composition and wave mode, emphasizing the need for experimental validation, extended frequency ranges, and shear wave investigations in future work. The ability to model both global energy loss through diffusivity and mechanistic damping via viscosity establishes a solid foundation for designing more accurate, non-invasive, and patient-tailored acoustic therapies. Ultimately, this work advances the scientific understanding required for the development of next-generation biomedical devices, such as the envisioned ultrasound-driven knee orthosis for cartilage regeneration, bringing us closer to therapeutic solutions that are not only effective, but biologically harmonious and clinically adaptable.

## 5. Conclusions

This study presents a comprehensive computational analysis of ultrasonic attenuation in biological tissues, integrating both sound diffusivity estimation and viscous damping models to better understand the underlying physical mechanisms. The experimental setup was considered sufficiently robust to measure the speed of sound, acoustic attenuation coefficient, and B/A nonlinearity parameter, yielding results consistent with those previously reported in the literature. By combining simulation with parameter estimation, the proposed framework offers a reproducible and computationally efficient method to obtain tissue-specific acoustic properties without relying solely on experimental setups. The computational results demonstrate that power-law regressions provide the most accurate representation of the frequency-dependent behavior of both sound diffusivity and bulk and dynamic viscosity, with the highest predictive power across a range of tissue types (R^2^ > 0.99 for most tissues). Although both the sound diffusivity and viscous attenuation models achieved high coefficients of determination in the time-domain validation (values), the sound diffusivity model more closely matched the experimental results in the frequency domain. Both approaches outperformed the linear solver in terms of accuracy, albeit at the cost of higher computational demand. Furthermore, regarding the viscous attenuation, it was found to be more sensitive to bulk viscosity than to dynamic viscosity, particularly in soft, fluid-rich tissues such as liver and myocardium, highlighting the dominant role of compressional damping in acoustic wave propagation. However, tissues such as cartilage and brain exhibited lower regression accuracy and minimal viscous response, suggesting the need for more complex modeling approaches, potentially incorporating poroelasticity, fractional viscoelasticity, or structural anisotropy to capture their behavior.

Moreover, the study emphasizes the importance of extending the frequency range in future research to include higher and intermediate frequencies, where additional attenuation mechanisms may emerge and power-law scaling can be further validated. It also highlights the need for shear wave-based investigations to accurately quantify dynamic viscosity, which remains undercharacterized in current models.

Future work should focus on extending the proposed modeling framework to in vivo validation, enabling assessment of its predictive accuracy under physiological conditions. In parallel, efforts should also be directed toward the integration of these models into clinical ultrasound systems, facilitating real-time, patient-specific parameter estimation to support both diagnostic imaging and therapeutic planning.

Altogether, these findings lay the groundwork for a more precise and physiologically grounded simulation framework capable of guiding the development of ultrasound-based therapeutic devices, such as non-invasive systems for cartilage regeneration. By combining phenomenological and mechanistic approaches, this work contributes to the broader goal of advancing personalized and targeted acoustic therapies, where modeling precision directly translates to clinical effectiveness and patient safety.

## Figures and Tables

**Table 1:** Overview of biological tissue samples evaluated in this study.

Biological Tissue	Origin	Number of Samples
Skin (thigh)	Porcine	5
Adipose Tissue (thigh)	Porcine	5
Skeletal Muscle (*biceps femoris*)	Porcine	10
Trabecular Bone (femur)	Bovine	5
Cortical Bone (femur)	Bovine	5
Liver	Bovine	7
Myocardium	Porcine	11
Kidney	Bovine	9
Tendon (*flexor digitorum medialis*)	Bovine	5
Ligament (anterior cruciate ligament)	Bovine	5
Cartilage (femorotibial joint)	Bovine	5
Brain (gray matter)	Porcine	7
Brain (white matter)	Porcine	7

**Table 2:** Number of mesh elements used in the simulation domain for each biological tissue sample.

Sample	Number of Domain Elements				
	0.5 MHz	1 MHz	2 MHz	4 MHz	5 MHz
Skin	4484	17,668	70,188	286,734	454,478
Adipose Tissue	3410	13,436	53,188	211,874	336,434
Skeletal Muscle (⟂ fibers)	4746	18,524	73,650	302,692	478,522
Skeletal Muscle (‖ fibers)	7502	29,298	117,240	500,168	782,262
Trabecular Bone	8484	33,556	133,310	567,116	904,214
Cortical Bone	4560	17,746	70,728	291,248	462,560
Liver	6412	25,404	101,128	427,390	681,504
Myocardium	4594	17,998	71,446	295,510	471,958
Kidney	4444	17,376	69,188	285,316	453,792
Tendon	3658	14,500	57,632	235,336	375,674
Ligament	2500	9766	39,208	155,366	243,538
Cartilage	1720	6786	26,426	104,988	165,692
Brain (gray matter)	5992	23,450	93,336	394,520	601,568
Brain (white matter)	5838	22,892	90,890	380,628	608,176

**Table 3:** Overview of the parameter estimation limits.

Parameters	Initial Value	Scale	Lower Bound	Upper Bound
Sound Diffusivity (m^2^/s)	0	1 × 10^−3^	0	0.2
Dynamic Viscosity (Pa·s)	0	1 × 10^−3^	0	0.2
Bulk Viscosity (Pa·s)	0	0.1	0	100

**Table 4:** Estimated power-law parameters for ultrasound attenuation in biological tissues.

Sample	$\alpha_{0}\;(dB/cm)$	*n*	$R^{2}$
Skin	1.096	1.157	0.9979
Adipose Tissue	0.7264	1.328	0.9989
Skeletal Muscle (⟂ fibers)	1.150	1.560	0.9983
Skeletal Muscle (‖ fibers)	2.870	1.458	0.9930
Trabecular Bone	1.784	1.525	0.9989
Cortical Bone	6.779	1.410	0.9999
Liver	0.5918	1.114	0.9998
Myocardium	0.5486	1.018	0.9968
Kidney	0.5115	1.016	0.9977
Tendon	4.617	1.123	0.9995
Ligament	1.489	1.180	0.9960
Cartilage	4.336	0.9553	0.9995
Brain (gray matter)	0.8513	0.9600	0.9986
Brain (white matter)	0.8862	1.026	0.9974

**Table 5:** Power-law regression parameters for sound diffusivity $\delta (f)\;=\;\delta_{0}\cdot f^{n}$ in biological tissues.

Sample	$\delta_{0}\;(m^{2}/s)$	*n*	$R^{2}$
Skin	0.0026	−0.934	0.9908
Adipose Tissue	0.0012	−0.608	1
Skeletal Muscle (⟂ fibers)	0.0024	−0.349	0.9980
Skeletal Muscle (‖ fibers)	0.0068	−0.379	0.9862
Trabecular Bone	0.0126	−0.364	0.9955
Cortical Bone	0.0837	−1.094	0.9965
Liver	0.0011	−0.842	1
Myocardium	0.0011	−0.936	1
Kidney	0.0010	−0.941	1
Tendon	0.0168	−0.801	0.999
Ligament	0.0038	−0.758	0.999
Cartilage	0.0261	−1.654	0.941
Brain (gray matter)	0.0017	−1.002	1
Brain (white matter)	0.0017	−0.932	1

**Table 6:** Power-law regression parameters for dynamic shear diffusivity in biological tissues.

Sample	$\mu_{0}\;(m^{2}/s)$	*n*	$R^{2}$
Skin	0.132	−0.454	0.9946
Adipose Tissue	0.019	−0.883	0.9975
Skeletal Muscle (⟂ fibers)	0.0071	−1.304	0.9685
Skeletal Muscle (‖ fibers)	0.0014	2.6616	0.9966
Trabecular Bone	0.0053	1.4635	0.998
Cortical Bone	0.2306	0.7249	0.9956
Liver	0.0219	−1.123	0.9938
Myocardium	0.0077	−1.077	0.9958
Kidney	0.0021	−1.572	0.9966
Tendon	0.0009	−0.588	0.9949
Ligament	0.0122	−0.814	0.9682
Cartilage	0.0526	−1.227	0.9997
Brain (gray matter)	0.0099	−0.501	0.9816
Brain (white matter)	0.0105	−0.506	0.9522

**Table 7:** Power-law regression parameters for bulk viscosity.

Sample	$\mu_{B,0}\;(m^{2}/s)$	*n*	$R^{2}$
Skin	3.0126	−0.794	1
Adipose Tissue	1.0874	−0.61	0.9999
Skeletal Muscle (⟂ fibers)	2.5212	−0.351	0.997
Skeletal Muscle (‖ fibers)	7.0265	−0.429	0.9963
Trabecular Bone	7.1265	−0.378	0.9973
Cortical Bone	238.62	−0.504	0.9931
Liver	1.1997	−0.846	0.9999
Myocardium	1.2287	−0.933	0.9999
Kidney	1.036	−0.929	0.9998
Tendon	18.626	−0.822	0.9987
Ligament	4.0994	−0.769	0.9999
Cartilage	12.646	−0.958	0.9992
Brain (gray matter)	1.7933	−1.007	1
Brain (white matter)	1.7447	−0.937	1

## Data Availability

All the necessary data are provided in this article.

## Abbreviations

The following abbreviations are used in this manuscript:

HIFU	High-Intensity Focused Ultrasound
LIPUS	Low-Intensity Pulsed Ultrasound
SoS	Speed of Sound
B/A	Non-Linearity Parameter

## Appendix A

This appendix presents all experimental results that were not included in the main Section 4.

### Appendix A.1

**Table A1:** Experimental values of the measured density of the biological samples.

Sample	Density (g/cm^3^)
Skin	1.13124 ± 0.05183
Adipose Tissue	0.927091 ± 0.01549
Skeletal Muscle	1.05775 ± 0.01573
Trabecular Bone	0.571999 ± 0.115192
Cortical Bone	1.86170 ± 0.10130
Liver	1.08368 ± 0.09560
Myocardium	1.09702 ± 0.08479
Kidney	1.05648 ± 0.03172
Tendon	1.12106 ± 0.04536
Ligament	1.10088 ± 0.05362
Cartilage	1.03345 ± 0.04336
Brain (gray matter)	1.04692 ± 0.01831
Brain (white matter)	1.02378 ± 0.02289

### Appendix A.2

**Table A2:** Experimental values of the measured speed of sound of the biological samples.

Sample	Speed of Sound (m/s)
Skin	1654.93 ± 32.82
Adipose Tissue	1461.77 ± 26.11
Skeletal Muscle (⟂ fibers)	1562.18 ± 20.76
Skeletal Muscle (‖ fibers)	1598.68 ± 34.34
Trabecular Bone	2325.38 ± 172.80
Cortical Bone	3193.95 ± 113.28
Liver	1539.69 ± 33.06
Myocardium	1585.83 ± 24.16
Kidney	1556.87 ± 17.38
Tendon	1827.43 ± 29.43
Ligament	1663.68 ± 42.29
Cartilage	1650.80 ± 51.95
Brain (gray matter)	1562.09 ± 53.55
Brain (white matter)	1538.46 ± 76.33

### Appendix A.3

**Table A3:** Measured ultrasound attenuation coefficients (*α*, in dB/cm) for biological tissues at five frequencies (0.5–5 MHz). Values are presented as mean ± standard deviation.

Sample	α (dB/cm)				
	0.5 MHz	1 MHz	2 MHz	4 MHz	5 MHz
Skin	0.521 ± 0.113	1.029 ± 0.217	2.356 ± 0.572	5.491 ± 1.118	7.323± 1.443
Adipose Tissue	0.301 ± 0.043	0.708 ± 0.161	1.721 ± 0.227	4.609 ± 0.814	6.391 ± 1.131
Skeletal Muscle (⟂ fibers)	0.397 ± 0.084	1.070 ± 0.264	3.663 ± 0.497	10.312 ± 1.650	13.432 ± 2.478
Skeletal Muscle (‖ fibers)	1.037 ± 0.181	2.894 ± 0.368	7.911 ± 0.802	21.647 ± 1.526	29.899 ± 1.970
Trabecular Bone	0.654 ± 0.105	1.679 ± 0.168	5.009 ± 0.457	14.641 ± 1.522	21.650 ± 2.652
Cortical Bone	2.562 ± 0.190	6.684 ± 0.844	18.335 ± 1.982	47.043 ± 6.160	66.284 ± 8.267
Liver	0.271 ± 0.007	0.604 ± 0.007	1.267 ± 0.020	2.754 ± 0.066	3.576 ± 0.074
Myocardium	0.273 ± 0.028	0.566 ± 0.116	1.021 ± 0.150	2.395 ± 0.303	2.785 ± 0.320
Kidney	0.242 ± 0.028	0.527 ± 0.109	1.104 ± 0.309	2.007 ± 0.591	2.600 ± 0.604
Tendon	2.119 ± 0.187	4.615 ± 0.464	10.161 ± 1.254	21.127 ± 2.563	28.890 ± 2.981
Ligament	0.608 ± 0.118	1.652 ± 0.357	3.405 ± 0.522	7.814 ± 0.838	9.405 ± 1.268
Cartilage	2.280 ± 0.536	4.214 ± 0.964	8.383 ± 2.327	16.593 ± 4.270	20.057 ± 5.292
Brain (gray matter)	0.454 ± 0.052	0.813 ± 0.095	1.643 ± 0.177	3.177 ± 0.338	4.119 ± 0.635
Brain (white matter)	0.412 ± 0.026	0.962 ± 0.099	1.801 ± 0.163	3.626 ± 0.352	4.567 ± 0.629

### Appendix A.4

**Table A4:** Average values and standard deviations of the B/A nonlinearity parameter for various biological tissues.

Sample	B/A Nonlinearity Parameter
Skin	7.237 ± 0.948
Adipose Tissue	10.754 ± 1.457
Skeletal Muscle (⟂ fibers)	6.632 ± 2.023
Skeletal Muscle (‖ fibers)	7.611 ± 1.099
Trabecular Bone	80.582 ± 21.027
Cortical Bone	12.948 ± 1.900
Liver	7.465 ± 0.197
Myocardium	7.188 ± 0.343
Kidney	7.045 ± 0.197
Tendon	7.047 ± 0.337
Ligament	7.206 ± 0.375
Cartilage	8.118 ± 0.227
Brain (gray matter)	7.216 ± 0.345
Brain (white matter)	7.476 ± 0.272

## Appendix B

This appendix presents all computational estimations that were not included in the main Section 4.

### Appendix B.1

**Table A5:** Calculated sound diffusivity values (δ) for various biological tissues.

Sample	δ (m^2^/s)				
	0.5 MHz	1 MHz	2 MHz	4 MHz	5 MHz
Skin	0.0046541	0.0026709	0.001542	0.000897	0.000446
Adipose Tissue	0.0018071	0.0011943	0.0007797	0.0005127	0.0004460
Skeletal Muscle (⟂ fibers)	0.0030714	0.0023591	0.0018314	0.0014533	0.0013849
Skeletal Muscle (‖ fibers)	0.0092088	0.0066373	0.0048843	0.0039815	0.0038835
Trabecular Bone	0.0165340	0.0123610	0.0094581	0.0075485	0.0071822
Cortical Bone	0.1680300	0.0835210	0.0438240	0.0223810	0.0112840
Liver	0.0020157	0.0011208	0.0006263	0.0003505	0.0002887
Myocardium	0.0021628	0.0011345	0.0005941	0.0003109	0.0002501
Kidney	0.0019224	0.0009979	0.0005220	0.0002731	0.0002189
Tendon	0.0293460	0.0165710	0.0095488	0.0060492	0.0043049
Ligament	0.0063957	0.0037370	0.0022007	0.0013106	0.0011161
Cartilage	0.1680300	0.0113610	0.0057624	0.0030086	0.0025893
Brain (gray matter)	0.0034649	0.0017210	0.0008619	0.0004319	0.0003438
Brain (white matter)	0.0032856	0.0017142	0.0008997	0.0004729	0.0003833

### Appendix B.2

**Table A6:** Calculated dynamic viscosity (μ) for various biological tissues.

Sample	μ (Pa·s)				
	0.5 MHz	1 MHz	2 MHz	4 MHz	5 MHz
Skin	0.018412	0.012715	0.010061	0.006913	0.006419
Adipose Tissue	0.035102	0.019314	0.010292	0.004623	0.005383
Skeletal Muscle (⟂ fibers)	0.016341	0.008656	0.002010	0.002483	0.000513
Skeletal Muscle (‖ fibers)	0.000194	0.001422	0.012017	0.052651	0.088172
Trabecular Bone	0.001883	0.005150	0.016389	0.038733	0.054621
Cortical Bone	0.129760	0.256180	0.380000	0.634210	0.714860
Liver	0.051783	0.020033	0.009566	0.004825	0.003678
Myocardium	0.017032	0.007693	0.003072	0.002192	0.001219
Kidney	0.006987	0.002228	0.000401	0.000297	0.000177
Tendon	0.001285	0.000902	0.000601	0.000419	0.000314
Ligament	0.021614	0.010881	0.007587	0.005932	0.002239
Cartilage	0.125650	0.051580	0.021970	0.009730	0.007340
Brain (gray matter)	0.014205	0.010266	0.006119	0.005506	0.004277
Brain (white matter)	0.016707	0.009774	0.006200	0.005904	0.004753

### Appendix B.3

**Table A7:** Calculated bulk viscosity ($\mu_{B}$) for various biological tissues.

Sample	$\mu_{B}$ (Pa·s)				
	0.5 MHz	1 MHz	2 MHz	4 MHz	5 MHz
Skin	5.247600	3.009100	1.722100	0.999730	0.845820
Adipose Tissue	1.668400	1.081400	0.709090	0.469090	0.407280
Skeletal Muscle (⟂ fibers)	3.261500	2.510400	1.931000	1.520400	1.480200
Skeletal Muscle (‖ fibers)	9.652800	6.910400	5.059100	4.033700	3.479900
Trabecular Bone	9.387300	7.118600	5.338900	4.135800	4.009300
Cortical Bone	329.040000	237.920000	180.340000	121.760000	99.465000
Liver	2.172700	1.187800	0.665870	0.373130	0.307230
Myocardium	2.356300	1.218700	0.645840	0.336810	0.273560
Kidney	1.979800	1.021700	0.551040	0.288260	0.229860
Tendon	32.594000	18.582000	10.578000	6.627900	4.496100
Ligament	7.002000	4.087400	2.419700	1.372100	1.218700
Cartilage	25.209000	12.351000	6.366900	3.334700	2.772700
Brain (gray matter)	3.608400	1.788100	0.893780	0.444900	0.353820
Brain (white matter)	3.340600	1.741900	0.912760	0.477130	0.385620

## Appendix C

This appendix presents all experimental results and simulation results of acoustic wave propagation that were not included in the text.
